# Vimentin network dysregulation mediates neurite deficits in *SNCA* duplication Parkinson’s patient–derived midbrain neurons

**DOI:** 10.1126/sciadv.adq2742

**Published:** 2025-06-06

**Authors:** Yanni Schneider, Aron Koller, Alina Schweigert, Marie Andert, Laura Hoffmann, Sydney O’Brien, Lukas Seebauer, Sonja Plötz, Pavel Kielkowski, Jürgen Winkler, Johannes C. M. Schlachetzki, Wei Xiang

**Affiliations:** ^1^ Department of Molecular Neurology, University Hospital Erlangen, Friedrich-Alexander-University Erlangen-Nürnberg, 91054 Erlangen, Germany.; ^2^ Department of Chemistry, Ludwig Maximilian University München, Würmtalstrasse 201, 81375 Munich, Germany.; ^3^ Department of Cellular and Molecular Medicine, University of California, San Diego, La Jolla, CA 92093, USA.; ^4^ Department of Neurosciences, University of California, San Diego, La Jolla, CA 92093, USA.

## Abstract

Duplication of the *SNCA* gene (*SNCA*^Dupl^), linked to elevated levels of α-synuclein (aSyn), is a genetic cause of Parkinson’s disease (PD). Our prior work with human-induced pluripotent stem cell (hiPSC)–derived midbrain neurons generated from patients with PD *SNCA*^Dupl^ identified neuritic deficits, accompanied by decreased levels of cytoskeletal element β-tubulin-III (bTubIII). To explore mechanisms underlying these effects in *SNCA*^Dupl^ neurons, we used CRISPR-Cas9 to generate isogenic control hiPSCs. Isogenic correction of *SNCA* dosage restored *SNCA*^Dupl^-induced neurite defects and bTubIII levels. Multi-omics analyses revealed *SNCA*^Dupl^-induced alterations in neuronal differentiation, with a notable down-regulation of PAX6. Moreover, *SNCA*^Dupl^ induced an up-regulation of vimentin. Further characterization revealed heightened vimentin truncation associated with altered distribution and organization. Similar changes in vimentin levels and truncation were observed in postmortem putamen tissue from patients with sporadic PD. Notably, targeting vimentin with okadaic acid and withaferin A restored bTubIII- and neurite-associated defects, suggesting its potential to prevent aSyn-mediated neuritic degeneration.

## Teaser

Find the effect of *SNCA* duplication on Parkinson’s neurons and how targeting *SNCA* and vimentin reverses neuronal deficits.

## INTRODUCTION

Parkinson’s disease (PD) is the most common neurodegenerative movement disorder, clinically characterized by motor symptoms, including bradykinesia, rigidity, and resting tremor. These symptoms are attributed to the progressive degeneration of midbrain dopaminergic neurons primarily located in the substantia nigra pars compacta. Neuropathologically, PD is defined by the presence of intracellular inclusions within neuronal bodies and neurites, so-called Lewy bodies (LB) and Lewy neurites (LN), respectively. Comprehensive investigations have identified α-synuclein (aSyn), especially in aggregated forms, as the major protein component in Lewy inclusions (*1*, *2*). Moreover, point mutations and the multiplication of the *SNCA* gene, which encodes aSyn, are associated with autosomal dominant forms of PD (*3*). Hence, both neuropathological and genetic evidence underscores the pivotal role of aSyn in the pathogenesis of PD.

Initially found in the electric ray *Torpedo californica*, aSyn was detected in the nucleus and nerve terminals of neurons (*4*). Because of its predominant localization in neurites, extensive research has been dedicated to understanding its role at the neuronal terminal. aSyn has been shown to modulate soluble *N*-ethylmaleimide–sensitive factor attachment protein receptor complex assembly (*5*), synaptic vesicle recycling (*6*, *7*), and neurotransmission (*8*, *9*). Coincidently, it has been implicated in the regulation of neurite development and function. Specifically, pathological forms of aSyn have been shown to impair axonal transport by interfering with cytoskeletal elements (*10*, *11*). These findings on the interplay between aSyn, neuritic, and synaptic functions are particularly intriguing, as a growing body of evidence suggests that neurodegeneration originates at the distal portion of neurites (*12*). However, the precise biological mechanisms underlying this interplay remain elusive.

Human-induced pluripotent stem cell (hiPSC)–derived neurons from patients with PD offer a unique opportunity to investigate PD-related aSyn pathology. Since an elevated level of aSyn is an established risk factor for PD pathology, the use of hiPSCs derived from patients with PD carrying *SNCA* gene multiplications has emerged as an important tool for modeling PD. Numerous studies, often using *SNCA* triplication hiPSC models, have revealed PD-associated cellular phenotypes such as mitochondrial dysfunction (*13*) and alterations in protein degradation pathways (*14*, *15*). Moreover, several studies on neurons derived from patients with PD carrying *SNCA* triplication have demonstrated impaired neurite morphology, vesicle transport deficits, and axonal degeneration compared to neurons from healthy individuals (*16*, *17*). Consistent with these findings, our own studies comparing neurons derived from patients with *SNCA* duplication (*SNCA*^Dupl^) PD with those from control donors, revealed altered neurite morphology (*18*) and axonal transport activity (*19*), along with disruption of microtubule organization (*18*). These alterations appeared to be linked to aSyn pathology: We observed increased aSyn accumulation along neurites (*20*) and interaction of aSyn with microtubule elements in *SNCA*^Dupl^ neurons (*18*).

Patients with PD harboring *SNCA*^Dupl^ or triplication have varying sizes of polygenic loci multiplicated in the genomic region of chromosome 4q22 (*21*). The rapid development of the CRISPR-Cas9–based genome editing tool provides a valuable tool for specifically modifying *SNCA* loci in cells derived from patients carrying *SNCA* multiplications. CRISPR-Cas9–mediated *SNCA* gene correction has been used in hiPSCs with *SNCA* triplication (*14*, *22*, *23*). This enables investigation into the specific effects of additional *SNCA* alleles without the influence of other multiplicated genes in the affected genomic regions.

In this study, we investigated whether the observed *SNCA*^Dupl^-mediated neurite impairments and cytoskeletal alterations in midbrain neurons are specific effects resulting from increased *SNCA* gene dosage. In addition, we explored the mechanisms underlying these *SNCA*^Dupl^-induced alterations. We reprogrammed fibroblasts from a female patient with PD carrying an *SNCA*^Dupl^ to hiPSCs. In addition, we generated an isogenic control from this *SNCA*^Dupl^ hiPSC line by restoring the *SNCA* gene expression. By comparing patient-derived *SNCA*^Dupl^ midbrain neurons with isogenic neurons, we revealed specific effects of elevated *SNCA* dosage. These effects include dynamic alterations in aSyn homeostasis during neuronal differentiation and impairments in neurite morphology. Using RNA sequencing (RNA-seq), assay for transposase-accessible chromatin sequencing (ATAC-seq), and proteomics, we identified that cellular pathways related to extracellular matrix (ECM) organization, neuronal differentiation, and synaptic function are significantly affected by increased *SNCA* dosage. In particular, *SNCA*^Dupl^ induced a remarkable up-regulation of vimentin. Further characterization revealed increased vimentin truncation, alongside its network disorganization and altered distribution along neurites in response to elevated *SNCA* dosage. Notably, by small molecule–mediated interference with the vimentin network, we were able to restore cytoskeletal and neuritic phenotypes related to *SNCA* gene duplication. Our findings shed light on an intrinsic interplay between aSyn and vimentin organization in neurons, thereby influencing cytoskeletal organization and neurite integrity.

## RESULTS

### *SNCA* duplication alters aSyn dynamics and distribution during neuronal differentiation

We obtained a skin biopsy from a 44-year-old female patient with PD with a genetically confirmed heterozygous duplication spanning an estimated region of 3.59 to 4.32 Mb on chromosome 4q22.1, which includes the *SNCA* gene. The patient began to develop PD-associated motor symptoms at age 39. In our previous study, we analyzed aSyn levels in neural precursor cells (NPCs) and midbrain neurons differentiated from hiPSCs, which were generated by reprogramming fibroblasts obtained from skin biopsies of this patient and healthy control donors (*18*). We observed significantly higher levels of aSyn in *SNCA*^Dupl^ NPCs and midbrain neurons at an early differentiation stage compared to their respective controls. These elevations were accompanied by neurite deficits and microtubule disorganization (*18*). To determine whether these phenotypes observed in *SNCA*^Dupl^ neurons are primarily attributable to *SNCA*^Dupl^ and whether correcting *SNCA* dosage can alleviate these effects, we used CRISPR-Cas9 technology to generate an isogenic hiPSC line (hereafter termed *SNCA*^Iso^) from the patient with *SNCA*^Dupl^ by inducing a heterozygous out-of-frame deletion of 118 base pair (bp) within exon 2 of *SNCA* [fig. S1, A to E, CRISPR-Cas9 procedure was described in more detail in (*24*)].

**Fig. 1.**
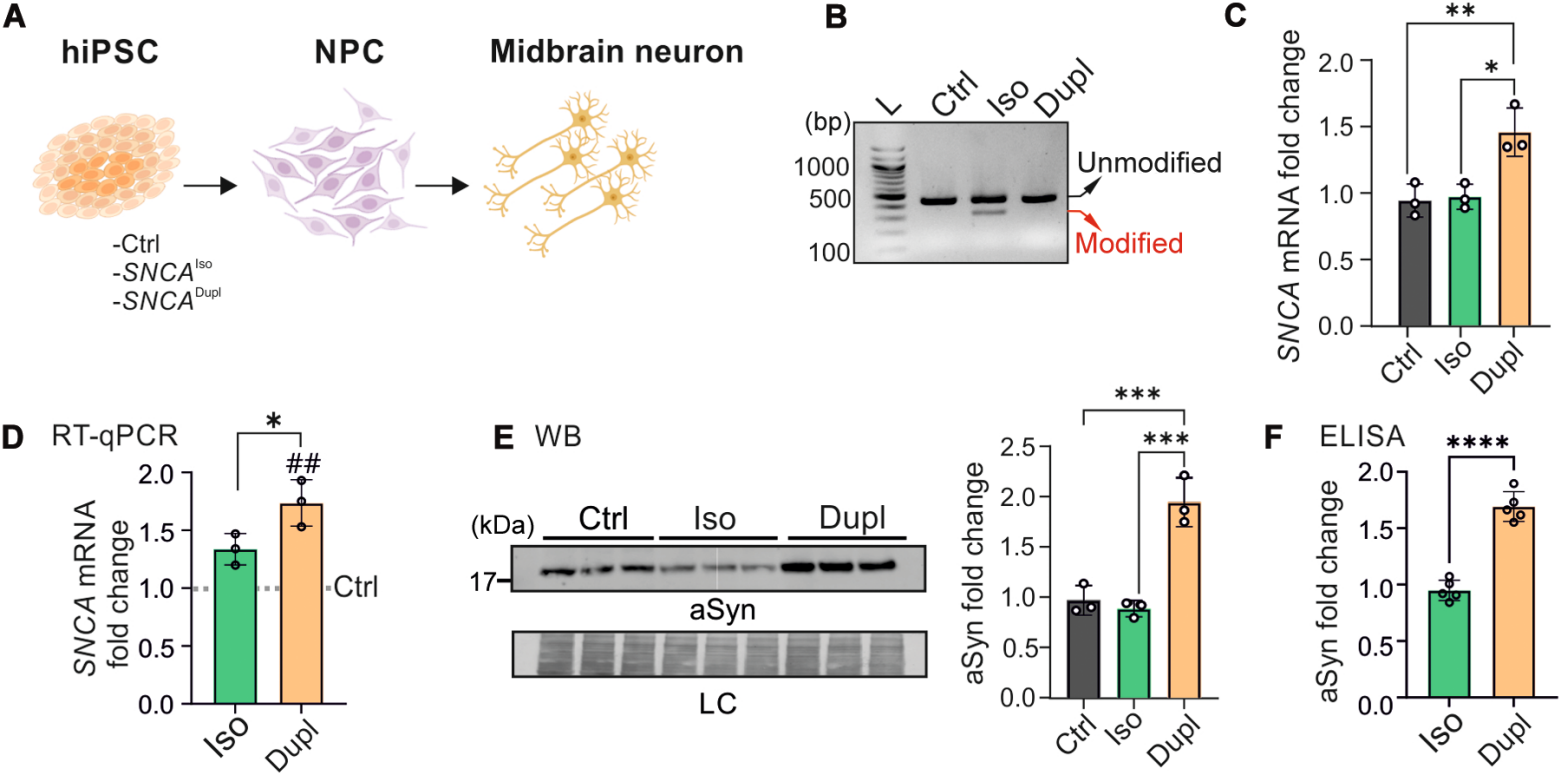
aSyn levels in *SNCA*^Dupl^ hiPSCs and NPCs. (**A**) Midbrain neuronal differentiation from hiPSC lines via NPCs. Created in BioRender. Xiang, W. (2025) https://BioRender.com/i9n38rh. (**B**) Agarose gel electrophoresis of PCR products. L, DNA ladder. The *SNCA*^Iso^ line displays a modified *SNCA* DNA fragment (~300 bp), alongside the unmodified fragment (~420 bp). (**C**) RT-qPCR analysis of *SNCA* mRNA, showing a higher level in the *SNCA*^Dupl^ compared to *SNCA*^Iso^ and control hiPSCs; *N* = 3 differentiation rounds. (**D** to **F**) RT-qPCR, WB, and ELISA analyses of *SNCA* mRNA (D) and aSyn protein [(E) and (F)] at the NPC stage. *SNCA* mRNA and aSyn levels are significantly higher in *SNCA*^Dupl^ NPCs than those in control [dashed line in (D)] and *SNCA*^Iso^ NPCs; *N* = 3 differentiation rounds. For (E), LC, Coomassie brilliant-blue staining. For (D), an additional unpaired *t* test was performed to compare *SNCA*^Iso^ or *SNCA*^Dupl^ with control NPCs, with ##*P* < 0.01.

Next, we investigated aSyn levels at different stages of neuronal differentiation, ranging from hiPSCs to midbrain neurons by comparing cells derived from *SNCA*^Dupl^, *SNCA*^Iso^, and control donors (Fig. 1A). We induced midbrain neuronal differentiation from hiPSCs via NPCs using a small molecule–based protocol (*18*, *25*). Following this protocol, neuronal differentiation yielded 80 to 90% neurons, as confirmed by flow cytometric analysis of the neuronal marker NeuN (fig. S1F). In addition, 30 to 50% of these neurons exhibited immunopositivity for both NeuN and tyrosine hydroxylase, indicative of dopaminergic neuron identity (fig. S1F). Immunocytochemistry (ICC) analysis using neuronal subtype–specific markers [tyrosine hydroxylase (TH) for dopaminergic neurons, glutamate decarboxylase 67 (GAD67) for GABAergic neurons, vesicular glutamate transporter 1 (vGlut1) for glutamatergic neurons, and choline acetyltransferase (ChAT) for cholinergic neurons] revealed that our midbrain cell model developed a small proportion of neurons positive for GAD67, vGlut1, and ChAT, while a larger proportion was positive for TH (fig. S1G).

At the hiPSC stage, polymerase chain reaction (PCR) amplification of the CRISPR-Cas9 targeted region followed by gel electrophoresis confirmed the presence of a modified *SNCA* DNA fragment with a deletion of approximately 100 bp in the isogenic hiPSC line (Fig. 1B). In addition, reverse transcription quantitative PCR (RT-qPCR) analysis revealed a 1.5-fold higher level of *SNCA* mRNA in the *SNCA*^Dupl^ line compared to the *SNCA*^Iso^ line and hiPSCs from a healthy control individual (Fig. 1C). Moreover, *SNCA* mRNA levels in the *SNCA*^Iso^ line were comparable to those of the control hiPSC line (Fig. 1C). These findings suggest heterozygous editing of *SNCA* and restoration of the *SNCA* dosage in the *SNCA*^Iso^ line.

To further analyze aSyn protein levels, hiPSCs were differentiated into NPCs. This step was necessary since aSyn expression is known to be induced during neuronal development (*26*, *27*), and full-length aSyn is absent in hiPSCs in our model (fig. S1D). At the NPC stage, we detected a significant increase in *SNCA* expression in *SNCA*^Dupl^ NPCs compared to *SNCA*^Iso^ or control NPCs, both at transcriptional and protein levels as determined by RT-qPCR (Fig. 1D) and Western blot (WB) (Fig. 1E), respectively. An additional enzyme-linked immunosorbent assay (ELISA) analysis revealed a 1.7-fold increase in aSyn levels in *SNCA*^Dupl^ compared to *SNCA*^Iso^ (Fig. 1F).

**Fig. 2.**
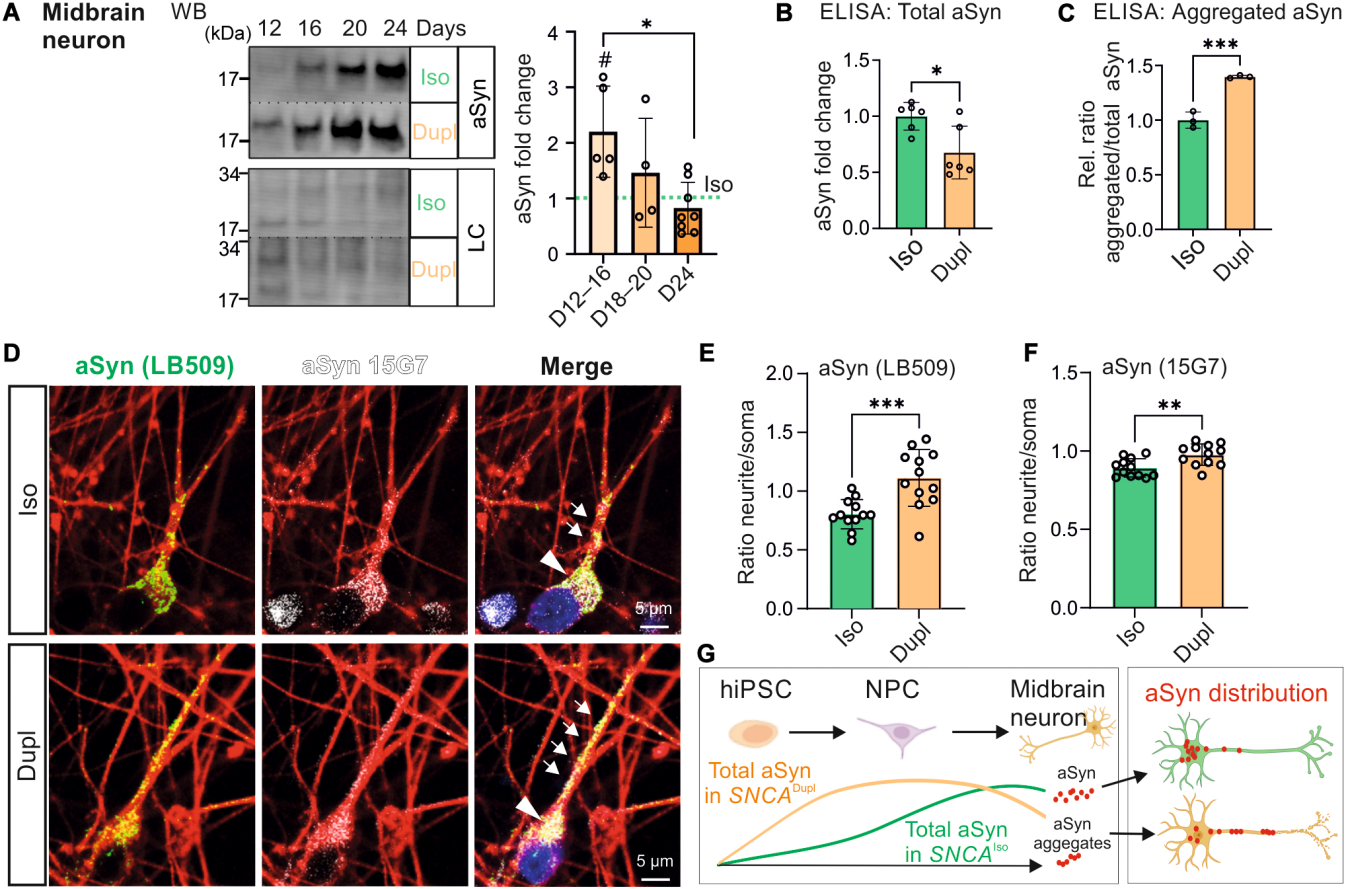
Dynamic aSyn expression during midbrain neuronal differentiation. (**A**) WB analysis of aSyn in *SNCA*^Dupl^ neurons during differentiation (12 to 24 days) compared to *SNCA*^Iso^ counterparts (green dashed line). aSyn levels are significantly higher in *SNCA*^Dupl^ neurons at days 12 to 16; however, the differences between *SNCA*^Dupl^ and *SNCA*^Iso^ neurons diminish over time. LC, revert 520 total protein stain; *N* = 4–8 differentiation rounds. An additional unpaired *t* test was performed to compare all *SNCA*^Dupl^ groups with their *SNCA*^Iso^ counterparts, with #*P* < 0.05. (**B**) ELISA analysis of total aSyn levels in neurons (24 days). *SNCA*^Dupl^ neurons display a significant lower aSyn levels than *SNCA*^Iso^ neurons. (**C**) ELISA analysis of the ratio of aggregated/total aSyn in neurons (24 days), demonstrating an increase in aggregated aSyn in *SNCA*^Dupl^ neurons. (**D** to **F**) ICC analysis of aSyn distribution using the 15G7 and LB509 antibodies, showing an increased ratio of intensity in neurite/soma in *SNCA*^Dupl^ compared to *SNCA*^Iso^ neurons, with a more pronounced effect observed using the LB509 antibody [(D) and (E)]. In (D), aSyn in the soma and along the neurite is highlighted with arrowheads and arrows, respectively. *n* = 12 neurons per group from *N* = 3 differentiation rounds. (**G**) Schematic summary of aSyn dynamics. Created in BioRender. Xiang, W. (2025) https://BioRender.com/yqichdf.

During the midbrain neuron differentiation, we assessed aSyn levels using WB analysis across early and late differentiation stages (Fig. 2, A to C, and fig. S2, A to B). Specifically, *SNCA*^Dupl^ neurons exhibited significantly higher aSyn levels during early differentiation (days 12 to 16) compared to *SNCA*^Iso^ neurons. These differences, however, became less pronounced as differentiation progressed to 24 days (Fig. 2A and fig. S2A). At day 24, *SNCA*^Dupl^ neurons exhibited even lower aSyn levels than *SNCA*^Iso^ neurons (Fig. 2A), as assessed by quantitative ELISA analysis (Fig. 2B). Similar trends were observed when comparing *SNCA*^Dupl^ neurons to healthy control neurons: aSyn levels were significantly elevated in *SNCA*^Dupl^ neurons at 10 days of differentiation compared to controls, but the differences declined by day 24 (fig. S2B).

To investigate whether *SNCA*^Dupl^ leads to a disturbance in aSyn homeostasis, we conducted an ELISA measuring the ratio of aggregated aSyn to total aSyn (Fig. 2C). This analysis revealed a significant, proportional increase of aggregated aSyn in *SNCA*^Dupl^ neurons compared to *SNCA*^Iso^ neurons.

We previously demonstrated increased aSyn accumulation along neurites in neurons from different patients with *SNCA*^Dupl^ compared to control individuals (*20*). Building upon this finding, we compared aSyn distribution in neurites and soma of *SNCA*^Dupl^ and *SNCA*^Iso^ midbrain neurons using ICC with two antibodies against aSyn: 15G7 and LB509 (Fig. 2, D to F). LB509 is frequently used to detect aggregated forms of aSyn (*28*), while the 15G7 antibody detects total aSyn. Consistent with our previous observations, we found increased accumulation of aSyn in neurites of *SNCA*^Dupl^ neurons compared to *SNCA*^Iso^ neurons. This was particularly evident with the LB509 antibody, indicating enhanced accumulation of aggregated aSyn in *SNCA*^Dupl^ neurites (Fig. 2, D and E).

In summary, *SNCA*^Dupl^ led to higher aSyn levels in NPCs and early differentiated neurons. However, as differentiation progressed, the difference in total aSyn levels between *SNCA*^Dupl^ and *SNCA*^Iso^ or control neurons diminished. Nevertheless, increased aSyn aggregation was observed in *SNCA*^Dupl^ neurons compared to controls, both in this study (*SNCA*^Dupl^ versus *SNCA*^Iso^) and in our previous study [*SNCA*^Dupl^ versus controls (*18*)]. In addition, *SNCA*^Dupl^ neurons exhibited increased accumulation of aSyn in neurites compared to controls [*SNCA*^Dupl^ versus *SNCA*^Iso^ in this study; *SNCA*^Dupl^ versus healthy control neurons in (*20*)]. Together, these collective findings suggest a spatially and temporally disturbed aSyn homeostasis in *SNCA*^Dupl^ neurons (summarized in Fig. 2G).

### *SNCA* duplication impairs neurite morphology and restored by corrected *SNCA* dosage

**Fig. 3.**
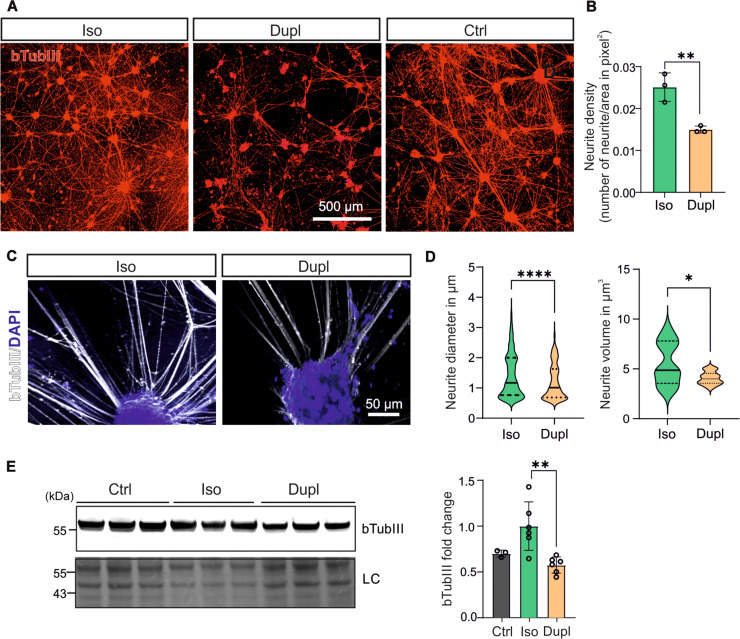
Analysis of neurites and bTubIII levels. (**A**) ICC analysis of the neuronal network using fluorescent staining for bTubIII, demonstrating a decrease in the number of neurites developed from *SNCA*^Dupl^ midbrain neurons compared to control and *SNCA*^Iso^ neurons. (**B**) Quantification of neurite density between *SNCA*^Iso^ and *SNCA*^Dupl^. (**C**) ICC images of bTubIII^+^ primary neurites (white) at higher magnification. (**D**) Analysis of the diameter and volume of bTubIII^+^ neurites using IMARIS, demonstrating a significant decrease in *SNCA*^Dupl^ neurons compared to *SNCA*^Iso^ neurons. For neurite diameter, *n* = 320–501 bTubIII^+^ neurites per group; for neurite volume, *n* = 70–900 bTubIII^+^ neurites per group from *N* = 3 differentiation rounds. (**E**) WB analysis of bTubIII levels. Left, a representative image; right, quantification of bTubIII showing lower levels in *SNCA*^Dupl^ neurons compared to control and *SNCA*^Iso^ neurons. LC, Ponceau staining. Quantitative data were derived from *N* = 3 differentiation rounds for control neurons and *N* = 6 differentiation rounds for *SNCA*^Dupl^ and *SNCA*^Iso^ neurons.

Our previous study comparing *SNCA*^Dupl^- and control individual–derived midbrain neurons demonstrated impaired neuritic morphology in neurons carrying *SNCA*^Dupl^ (*18*). This was accompanied by reduced levels and reorganization of β-tubulin-III (bTubIII), a crucial structural component of neuronal microtubules. To investigate whether these observed effects are specifically attributable to *SNCA*^Dupl^, we performed ICC analysis of bTubIII and found an overall impaired neuritic network characterized by remarkably lower neurite density in *SNCA*^Dupl^ neurons compared to *SNCA*^Iso^ neurons (Fig. 3, A and B) and those from control donors [Fig. 3A and also characterized in (*18*)]. Isogenic correction of *SNCA*^Dupl^ restored this neurite phenotype to a level comparable to that of control neurons (Fig. 3A). Further analyses revealed that neurites of *SNCA*^Dupl^ neurons exhibited a significant decrease in diameter and volume compared to those of *SNCA*^Iso^ neurons (Fig. 3, C and D, and fig. S3, A and B). In addition, we assessed cell death in *SNCA*^Iso^ and *SNCA*^Dupl^ neurons using the lactate dehydrogenase cytotoxicity assay without detecting significant differences (fig. S3C). These findings suggest that the neurite deficits observed in *SNCA*^Dupl^ neurons are not due to extensive neuronal loss, highlighting neurite degeneration as an early event preceding neuronal loss. Coinciding with neurite deficits, bTubIII levels were lower in *SNCA*^Dupl^ compared to control midbrain neurons, and bTubIII levels were restored in *SNCA*^Iso^ neurons (Fig. 3E).

Together, our results indicate that the altered neuronal network, including structural changes associated with neurite development and bTubIII changes observed in *SNCA*^Dupl^ midbrain neurons are specific outcomes of *SNCA* gene duplication during neuronal differentiation. In addition, correcting *SNCA* dosage reverses the neurite phenotypes and bTubIII levels in *SNCA*^Dupl^ midbrain neurons.

### *SNCA* duplication changes gene expression affecting neuronal differentiation and function

**Fig. 4.**
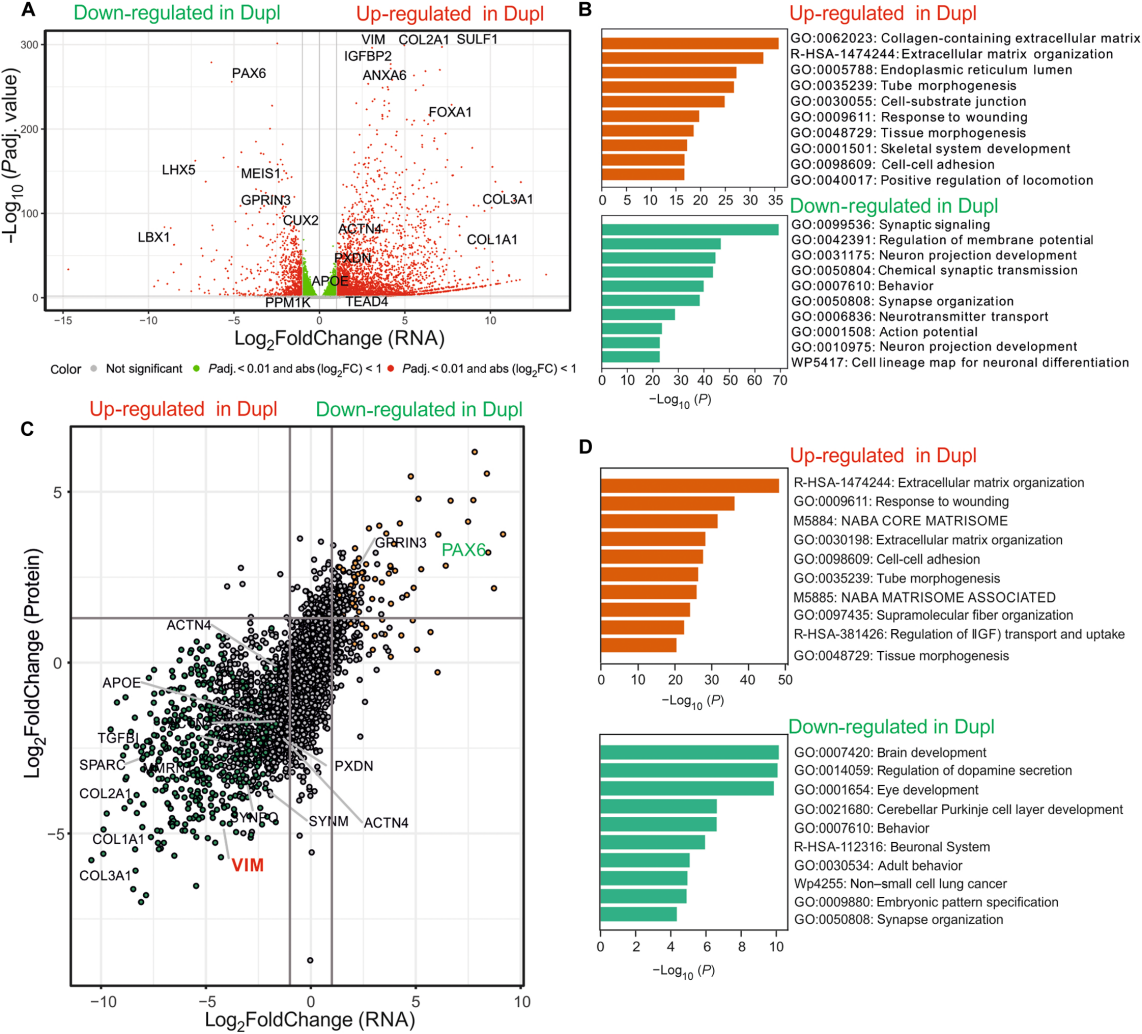
Transcriptomic and proteomic analyses. (**A**) Volcano plot of differentially expressed genes identified by transcriptomics via RNA-seq comparing *SNCA*^Dupl^ to *SNCA*^Iso^ midbrain neurons. Thresholds for log_2_-fold changes were set to 1 and −1 (gray lines). Transcript annotation was performed using the ggrepl package in R. Significantly up- (right) and down-regulated (left) genes are highlighted in red. *X* axis, log_2_-fold change of the mean expression levels; *y* axis, negative decade logarithm of the *P* value [−log_10_ (*P* value)]. (**B**) Gene ontology pathway analysis of differentially regulated genes. Top 10 pathways for up- (top) and down-regulated genes (bottom) in *SNCA*^Dupl^ midbrain neurons are displayed. For (A) and (B), *N* = 2 differentiation rounds, with experimental duplicates per round. (**C**) Correlation analysis of proteomic and transcriptomic hits based on Pearson correlation coefficient. The analysis includes 421 differentially expressed proteins and mRNAs with at least a twofold change (log_2_ <−1 or >1) and an adjusted *P* value <0.05. A significant correlation is observed (*r*^2^ = 0.64, *P* = 2.69 × 10^−49^) between the transcriptomic and proteomic hits. VIM as one of the most up-regulated elements and PAX6 as one of the most down-regulated elements are highlighted. (**D**) Gene ontology pathway analysis of differentially expressed features both at protein and mRNA levels in *SNCA*^Dupl^ compared to *SNCA*^Iso^ midbrain neurons. Top 10 pathways for commonly up-regulated (top) and down-regulated features (bottom) in *SNCA*^Dupl^ midbrain neurons are depicted. *X* axis, adjusted – log_10_ *P* value; *y* axis, top 10 identified pathways for up-regulated (top) or down-regulated features (bottom). For (C) and (D), *N* = 2 differentiation rounds for *SNCA*^Dupl^ midbrain neurons and *N* = 3 differentiation rounds for *SNCA*^Iso^ midbrain neurons, with experimental duplicates per round.

On the basis of the observed alterations in aSyn dynamics and neurite deficits mediated by *SNCA*^Dupl^, we sought to explore underlying mechanisms using multiple omics approaches. Since similar patterns were observed in these phenotypes between *SNCA*^Iso^ and control cells, and to dissect the specific effects of *SNCA*^Dupl^, we focused on comparisons between *SNCA*^Dupl^ and *SNCA*^Iso^ midbrain neurons. We performed transcriptomic analysis using RNA-seq and found extensive alterations in *SNCA*^Dupl^ neurons, evident by the identification of 3011 up-regulated and 909 down-regulated transcripts (adjusted *P* value <0.01 and fold change >2) compared to *SNCA*^Iso^ neurons (Fig. 4A). Gene ontology analysis of significantly up-regulated transcripts in *SNCA*^Dupl^ neurons further revealed enrichment in genes associated with ECM-related activities, such as ECM organization and cell-cell adhesion (Fig. 4B, top). Notably, *VIM* (encoding for vimentin) was identified as one of the most up-regulated genes in *SNCA*^Dupl^ midbrain neurons. Vimentin is an element of intermediate filaments, a crucial component of the cytoskeleton, and is involved in the modulation of signaling cascades that regulate ECM modeling (*29*). Consistent with alterations in ECM, genes encoding several collagen isoforms were found to be up-regulated in *SNCA*^Dupl^ midbrain neurons (Fig. 4A). In addition, we observed the down-regulation of genes associated with neuronal maintenance and function in *SNCA*^Dupl^ neurons, consistent with the observed neurite deficits. Enriched pathways included synaptic signaling, synapse organization, neuronal projection, and neuronal differentiation (Fig. 4B, bottom). Regarding neuronal differentiation, PAX6 was identified as one of the most down-regulated genes. In addition, *LHX5*, a member of the LHX family of transcription factors, which is involved in brain development, was found to be another gene among the most down-regulated genes in *SNCA*^Dupl^ neurons (Fig. 4A). *GPRIN3*, a member of the G protein–regulated inducer of neurite outgrowth family known for its regulatory role in dopamine receptor signaling (*30*), exhibited significant down-regulation in *SNCA*^Dupl^ midbrain neurons (Fig. 4A and fig. S4A). *GPRIN3* is situated adjacent to the *SNCA* locus and is also present within the heterozygous duplicated locus in *SNCA*^Dupl^ cells. As expected, CRISPR-Cas9–mediated gene editing of *SNCA* (*SNCA*^Iso^) did not initially affect the gene expression of *GPRIN3*, indicated by consistently elevated mRNA levels, determined using RT-qPCR in both *SNCA*^Iso^ and *SNCA*^Dupl^ NPCs compared to control NPCs (fig. S4A). During midbrain neuronal differentiation, however, *GPRIN3* expression is substantially down-regulated in *SNCA*^Dupl^ neurons, implying its role in neuronal differentiation. This observation also highlights the impact of increased *SNCA* dosage on the dynamic regulation of *GPRIN3* expression.

Next, we conducted a proteomic analysis of *SNCA*^Dupl^ and *SNCA*^Iso^ midbrain neurons. Consistent with the transcriptomic results, we observed increased protein levels of vimentin and proteins crucial for ECM activity, such as collagen isoforms, alongside decreased protein levels of neuronal differentiation–associated transcription factors like PAX6, LHX5, and GPRIN3 (fig. S4B). This prompted us to investigate whether changes in gene transcription are reflected in protein abundance. We compared differentially regulated transcripts and proteins, exhibiting changes of log_2_ <−1 or >1 fold with an adjusted *p* value <0.05. We identified 425 up-regulated and 60 down-regulated features that are changed in *SNCA*^Dupl^ neurons at both mRNA and protein levels, displaying a robust correlation of Pearson = 0.64 [*P* = 2.69 × 10^−49^; 95% confidence interval (0.58. 0.69), Fig. 4C]. Gene ontology analysis using these features regulated at both mRNA and protein levels confirmed the enrichment of ECM-associated processes (such as ECM organization and cell-cell adhesion) specifically through *SNCA* dosage enhancement, while processes associated with the nervous system (such as brain development and synapse organization) were down-regulated (Fig. 4D). In summary, through a combined transcriptomic and proteomic analysis, we established a robust correlation between gene and protein expression levels, highlighting the impact of *SNCA* dosage on cellular processes related to neuronal differentiation, function, and cellular structural integrity.

### *SNCA* duplication alters the chromatin accessibility landscape

*SNCA* duplication resulted in substantial changes in the gene expression and proteome of midbrain neurons, but the molecular mechanisms underlying these alterations remain incompletely understood. Since gene expression is tightly regulated by chromatin accessibility, we performed ATAC-seq to investigate changes in the chromatin accessibility landscape. To further identify transcriptional regulators driving gene expression, we conducted motif enrichment analysis of open chromatin regions to infer key transcription factors driving gene expression during development mediated by *SNCA* duplication.

**Fig. 5.**
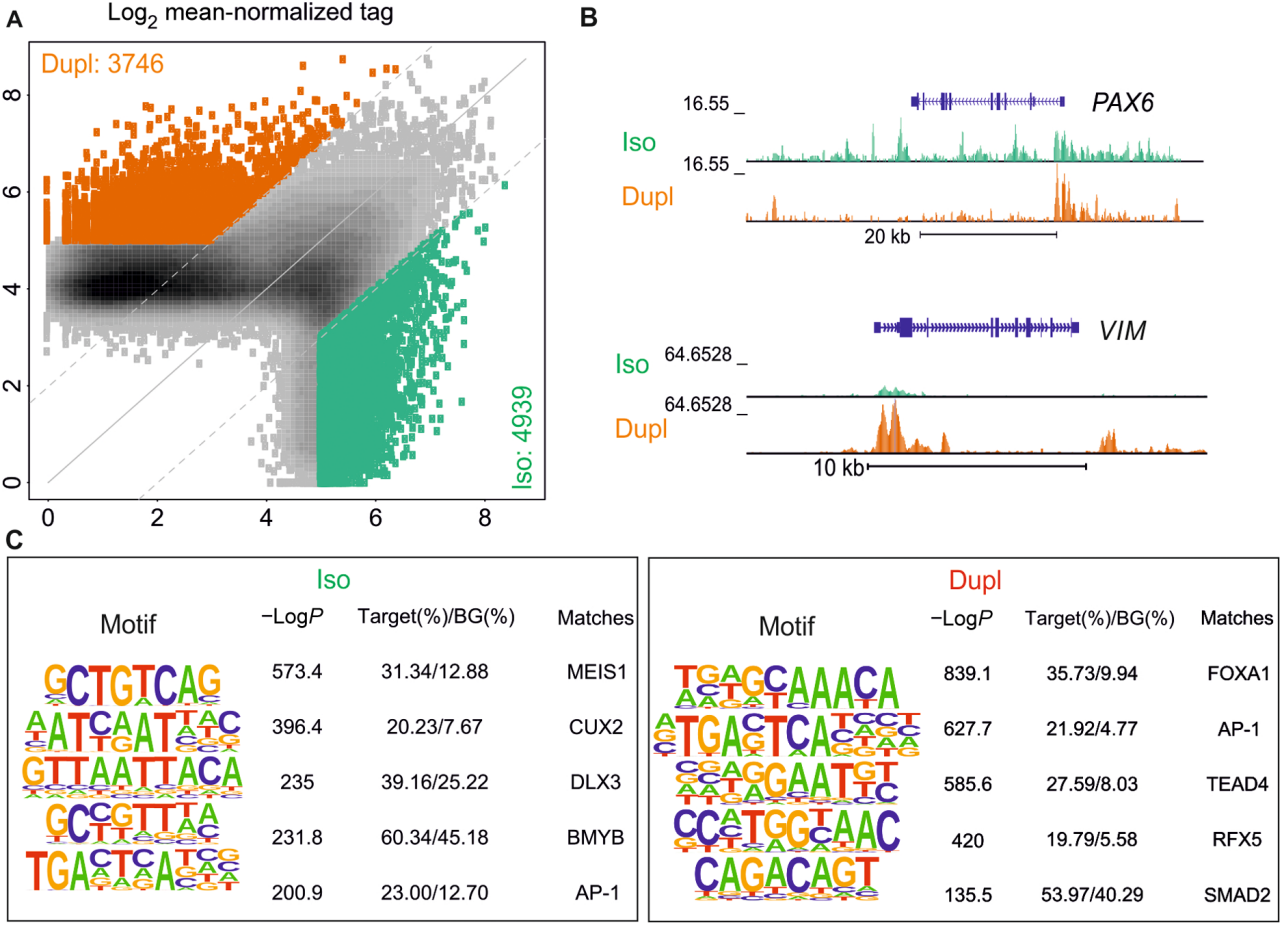
Analysis of chromatin accessibility and transcription factor motif enrichment using ATAC-seq. (**A**) Scatterplot of ATAC-seq peaks in *SNCA*^Dupl^ versus *SCNA*^Iso^ neurons. Color codes indicate significant changes (orange and green are fold change >2 and <−2, respectively, *P*adj <0.05, calculated from DESeq2 analysis). (**B**) Genome browser tracks of ATAC-seq of *SNCA*^Dupl^ (orange) and *SNCA*^Iso^ (green) neurons at the PAX6 and VIM loci. (**C**) De novo motif analysis of ATAC-seq peaks at regions of open chromatin. Target (%) is the number of target sequences with motifs over total target sequences; %BG (%background) is the number of background sequences with motif over total background sequences. Data were derived from *N* = 2 differentiation rounds, with experimental duplicates/round. *P* values were calculated using binomial distribution in HOMER.

Analysis of ATAC-seq data indicated that *SNCA*^Dupl^ was associated with a >2-fold increase in ATAC signal at 3476 and a >2-fold decrease at 4939 distal sites (>±3 kb from transcription start site; adjusted *P* value <0.05) in midbrain neurons (Fig. 5A). As two examples of affected genes, *PAX6* demonstrated decreased open chromatin regions, while *VIM* exhibited increased open chromatin regions in *SNCA*^Dupl^ midbrain neurons (Fig. 5B), consistent with their altered expression levels identified through transcriptomic analysis (Fig. 4A).

Additional gene ontology analysis of genes exhibiting differential chromatin accessibility between *SNCA*^Dupl^ and *SNCA*^Iso^ neurons revealed enrichment of genes associated with various aspects of nervous system development, such as head development and regulation of nervous system development as well as pathways related to neuronal functions like synaptic signaling. In addition, genes involved in cellular structural elements, including cell morphogenesis and cell-cell adhesion, were found to be enriched (fig. S5). Notably, the enriched pathways exhibit notable similarities to pathways found in transcriptomics and proteomics (Fig. 4, B and D). This implies an interplay between expression regulation and chromatin accessibility in those observed biological processes.

To identify key transcription factors underlying the observed changes in gene expression between *SNCA*^Dupl^ and *SNCA*^Iso^ midbrain neurons, we performed de novo motif analysis of differentially regulated enhancers (Fig. 5C). One common transcription factor–binding motif identified was associated with AP-1. This AP-1–binding motif is highly enriched in open chromatin regions exhibiting both increased and reduced accessibility in *SNCA*^Dupl^ compared to *SNCA*^Iso^ midbrain neurons. AP-1 is known to be involved in various neuronal functions including cell growth, differentiation, survival, and synaptic plasticity (*31*). Moreover, the most significantly enriched sequences in *SNCA^Dupl^* corresponded to motifs recognized by several additional transcriptional factors like FOXA1, RFX5, and SMAD2. These transcription factors have been associated with neuronal development and survival (*32*), development and maintenance of specific neuronal subtypes (*33*, *34*), as well as neuronal function (*32*). In contrast, binding motifs for MEIS1 and CUX2 were enriched in peaks reduced or lost in *SNCA*^Dupl^ compared to *SNCA*^Iso^ midbrain neurons. These transcription factors are also implicated in neuronal differentiation and brain development (*35*, *36*). In summary, *SNCA* duplication in midbrain neurons induced significant alterations in chromatin accessibility and usage of transcription factors involved in neuronal development and synaptic signaling.

### *SNCA* duplication decreases PAX6 expression during neuronal differentiation

**Fig. 6.**
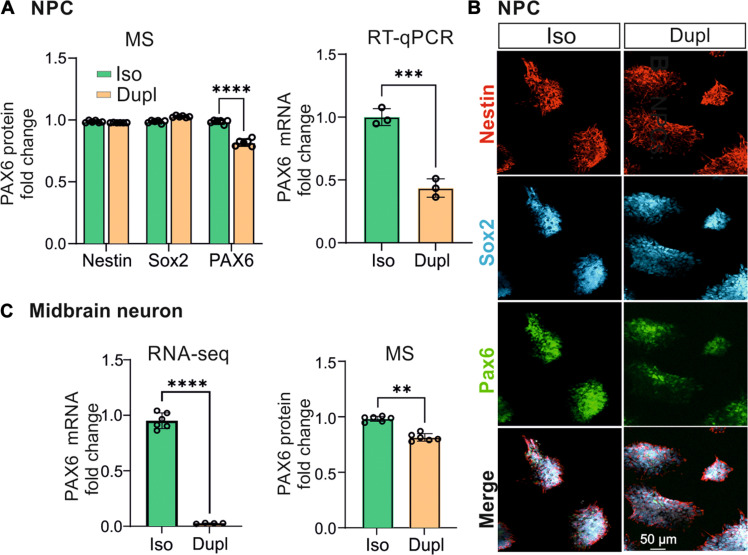
PAX6 expression. (**A**) Left, MS protein analysis of Nestin, Sox2, and PAX6, demonstrating unchanged levels of Nestin and Sox2 between *SNCA*^Dupl^ and *SNCA*^Iso^ NPCs. However, PAX6 levels are significantly reduced in *SNCA*^Dupl^ NPCs. Right, RT-qPCR analysis of *PAX6* mRNA, showing a significant down-regulation in *SNCA*^Dupl^ NPCs. (**B**) ICC analysis of NPC markers Nestin (red), Sox2 (cyan), and PAX6 (green) in NPCs. (**C**) Left, MS analysis of PAX6 protein levels; right, RNA-seq analysis of *PAX6* mRNA levels in differentiated midbrain neurons. Both display a significant decrease in *PAX6* gene and protein levels in *SNCA*^Dupl^ neurons. For (A) left and (C), *N* = 2–3 differentiation rounds for *SNCA*^Dupl^ midbrain neurons and *N* = 3 differentiation rounds for *SNCA*^Iso^ neurons, with *n* = 2 technical replicates. For (A) right, *N* = 3 independent differentiation rounds. Statistics in [(A), left], two-way analysis of variance (ANOVA) with Šídák’s multiple comparisons.

Our combined multi-omics approaches unveiled alterations in neuronal differentiation induced by *SNCA*^Dupl^, characterized particularly by a down-regulation of PAX6 expression (Fig. 4). Since PAX6 is a well-known NPC marker, we examined its expression at the NPC stage. WB analysis using an antibody targeting a 17–amino acid PAX6 peptide showed no significant difference in PAX6 levels between *SNCA*^Dupl^ and *SNCA*^Iso^ NPCs (fig. S6A). However, mass spectrometric (MS) analysis for detecting various proteoforms across the entire protein sequence, demonstrated a significant reduction in PAX6 protein expression (Fig. 6A, left). Supporting this finding, ICC analysis showed a significant decrease in PAX6 fluorescence intensity (Fig. 6B and fig. S6B), and flow cytometry revealed a smaller PAX6-immunoreactive population (fig. S6C) in *SNCA*^Dupl^ NPCs compared to *SNCA*^Iso^ NPCs. Consistently, RT-qPCR further confirmed reduced PAX6 expression at the mRNA level (Fig. 6A, right). Among crucial transcriptional factors involved in neuronal development and differentiation into mature neurons, such as Nestin, Sox2, and PAX6 (*37*–*39*), which were all expressed in both *SNCA*^Iso^ and *SNCA*^Dupl^ NPCs (Fig. 6B), only PAX6 exhibited a significant reduction in *SNCA*^Dupl^ NPCs as assessed by MS analysis (Fig. 6A, left).

Notably, the down-regulation of PAX6 expression persisted during midbrain neuronal differentiation. Both MS and WB demonstrated decreased PAX6 protein levels (Fig. 6C, right, and fig. S6A), while RNA-seq analysis and RT-qPCR confirmed reduced PAX6 mRNA expression (Fig. 6C, left, and fig. S6D). In total, multiple complementary approaches revealed a significant reduction in PAX6 expression in *SNCA*^Dupl^ cells, both at the mRNA and protein level, as well as in both NPCs and differentiated midbrain neurons.

### *SNCA* duplication disrupts vimentin levels, truncation, organization, and neurite distribution

Our unbiased multi-omics analyses identified vimentin as one of the most significantly differentially expressed proteins. Vimentin, an integral component of the cytoskeleton, is involved in ECM organization, regulating collagen expression and release [reviewed in (*29*)], as well as in neuronal differentiation (*40*) and neurite development (*39*). Since these cellular processes are particularly affected in *SNCA*^Dupl^ midbrain neurons according to pathway enrichment analyses (Fig. 4, B and D), we proceeded to validate the effects of *SNCA*^Dupl^ on vimentin biology compared to *SNCA*^Iso^ neurons.

**Fig. 7.**
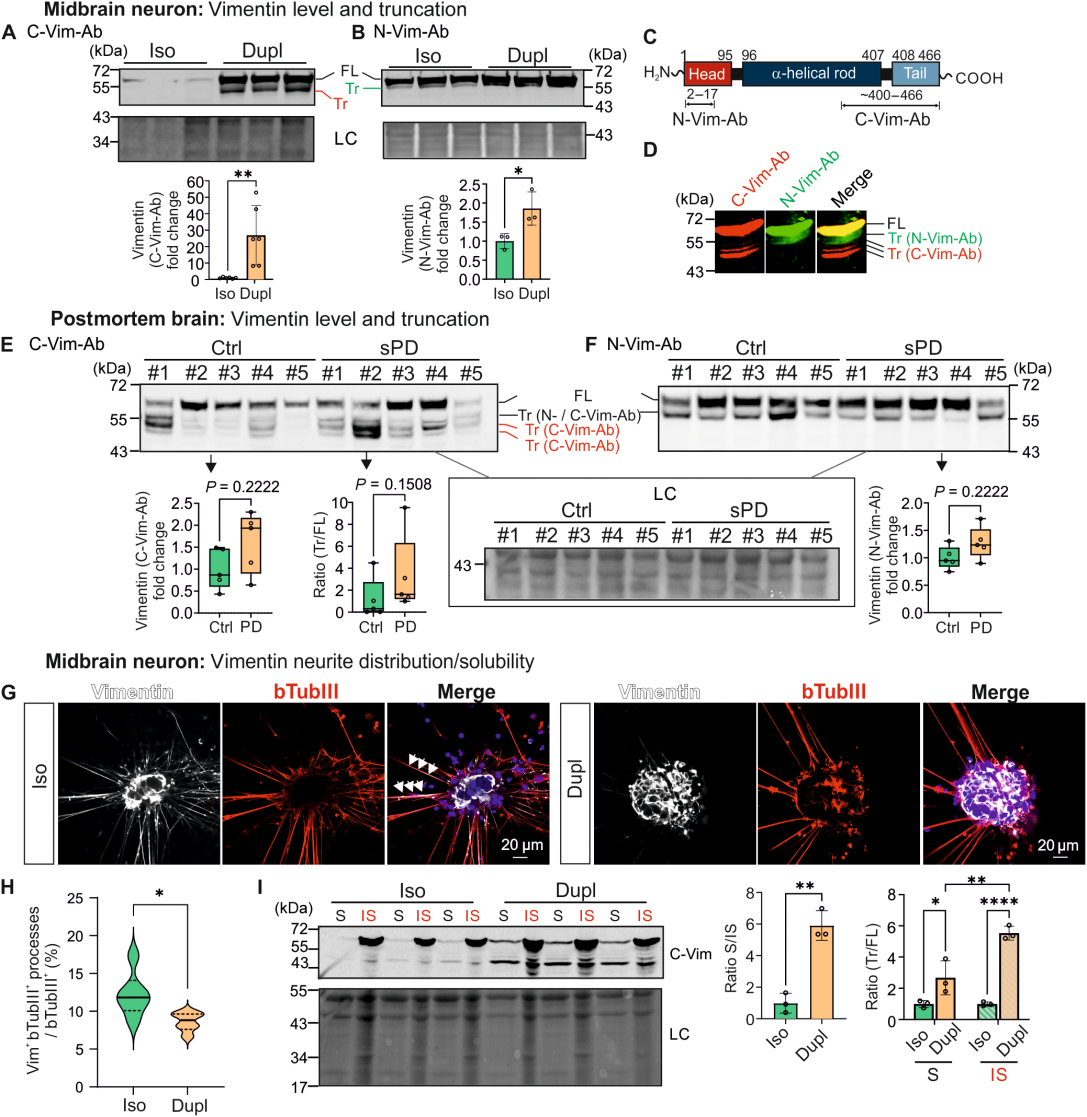
Vimentin expression, truncation, subcellular localization, and organization. (**A** and **B**) WB analysis of vimentin using the C-terminal [C-Vim-Ab, (A)] and the N-terminal [N-Vim-Ab, (B)] vimentin antibodies. Vimentin levels are significantly increased in *SNCA*^Dupl^ neurons compared to *SNCA*^Iso^ neurons. Notably, the levels of truncated vimentin <55 kDa (Tr, red) is increased in *SNCA*^Dupl^ neurons (A). LC, Ponceau staining. For (A), *N* = 6; for (B) *N* = 3 differentiation rounds. (**C**) Vimentin amino acid sequence and epitopes targeted by the N- and C-terminal antibodies. (**D**) WB image of a *SNCA*^Dupl^ sample, probed with the C- (red) or N-terminal (green) vimentin antibody, following SDS-PAGE with extended run time. Truncated vimentin forms are detected by C- and N-terminal antibodies and highlighted in green and red, respectively. (**E** and **F**) WB analysis of vimentin in the putamen of patients with sporadic PD and controls using C- and N-terminal vimentin antibodies. Truncated forms detected by the C-terminal antibody only are highlighted in red. LC, Ponceau staining. Both antibodies detected a trend toward increased vimentin levels in sporadic PD. Additional quantification of the ratio of truncated/full-length vimentin [(E) bottom right] demonstrates a trend toward elevated truncation in sporadic PD. *N* = 5 per group. (**G**) ICC images of vimentin (white) and bTubIII (red). Arrows highlight vimentin^+^ and bTubIII^+^ neurites. (**H**) Quantification of vimentin^+^ and bTubIII^+^ double-positive neurites among all bTubIII^+^ neurite, demonstrating a significant decrease in neuritic vimentin distribution in *SNCA*^Dupl^ neurons compared to *SNCA*^Iso^ neurons. *n* = 6 individual cells/group of *N* = 3 differentiation rounds. (**I**) Analysis of vimentin solubility using a solubility assay followed by WB analysis of vimentin using the C-terminal vimentin antibody. *SNCA*^Dupl^ neurons show a significant increase in the ratio of soluble (S)/insoluble (IS) vimentin and an overall higher amount of truncated vimentin forms in both soluble and insoluble fractions, compared to *SNCA*^Iso^ neurons. LC, Ponceau staining. *N* = 3 differentiation rounds.

Firstly, we validated vimentin expression levels via WB analysis, using two different antibodies targeting C-terminal and distal N-terminal regions of the protein (Fig. 7, A to C). We confirmed markedly elevated vimentin levels in *SNCA*^Dupl^ midbrain neurons using both antibodies, although the determined fold changes varied. These differences may result from variations in antibody binding affinity, potentially influenced by distinct posttranslational modification patterns of vimentin in hiPSC-derived neurons. The significant increase in vimentin expression in *SNCA*^Dupl^ neurons was already evident at the NPC stage (fig. S7A). Intriguingly, we detected a vimentin band below 55 kDa, which was exclusively recognized by the C-terminal antibody and showed a significant increase in intensity (Fig. 7A). Extending the SDS–polyacrylamide gel electrophoresis (SDS-PAGE) run time resolved several additional vimentin-related bands below the full-length vimentin band, which were detected by either N- or C-terminal vimentin antibodies, suggesting the presence of various truncated forms (Fig. 7D and fig. S7B). Since the truncated forms below 55 kDa were barely detected in *SNCA*^Iso^ neurons and increased in *SNCA*^Dupl^ (Fig. 7A), we focused on the characterization of these forms in the following analyses. We conducted MS analysis following in-gel digestion of full-length vimentin (466 amino acids) and the <55-kDa truncated forms (fig. S7C and table S5). Consistent with their absence when using the distal N-terminal antibody, MS revealed the missing N-terminal fragment ^13^RMFGGPGTASRPSSSR^28^ in the truncated forms, indicating that the N-terminal truncation involves this fragment. Furthermore, MS identified the loss of the distal C-terminal fragment ^451^DGQVINETSQHHDDLE^466^. Despite the absence of this most C-terminal fragment, the C-terminal antibody can still detect truncated vimentin, assuming the binding to a broader region within the C-terminal domain.

To assess the pathological relevance of these observations on vimentin in synucleinopathies, we extended the vimentin analysis to human postmortem putamen tissue derived from patients with sporadic PD and controls. Despite the limited sample size (*n* = 5 per group), we noted a trend toward increased total vimentin levels in patients with PD using both vimentin antibodies (Fig. 7, E to F, left and right), in line with our findings in hiPSC-derived *SNCA*^Dupl^ midbrain neurons. Moreover, WB analysis revealed at least three different vimentin truncation bands (Fig. 7E) in human putamen tissue. These forms were detected either by both antibodies or only by the C-terminal antibody, with higher levels observed in patients with PD (Fig. 7E, middle quantification). In addition, we analyzed vimentin in mouse models of PD and multiple system atrophy (MSA), overexpressing human aSyn either under the Thy-1 promoter [Thy1-*SNCA*, line 61, (*41*)] or the myelin basic protein promoter [MBP*-SNCA*, line 29, (*42*)], respectively. In both synucleinopathy mouse models, we observed significantly elevated vimentin levels in brain tissue compared to their respective nontransgenic counterparts (fig. S7, D and E). Increased vimentin truncation was also observed in synucleinopathy mouse brains, detected by the N-terminal antibody. The discrepancy in the sensitivity of vimentin antibodies to detect truncated forms in different models, such as hiPSC-derived neurons, postmortem PD brains, and synucleinopathy mouse models, may result from variations in truncation sites, protease involvement, and the degree of vimentin cleavage in each system. These factors likely lead to differential exposure of C-terminal or N-terminal epitopes, which are recognized by the respective antibodies. Despite these discrepancies in truncation detection, our collective data from different models point to an interplay between aSyn overexpression and increased vimentin expression and truncation.

Given the pronounced neuritic phenotypes in *SNCA*^Dupl^ midbrain neurons, we proceeded to analyze vimentin distribution in neuritic processes by ICC analysis. We observed a notable decrease in vimentin-positive neurites despite increased protein expression, with vimentin accumulating in somata (Fig. 7, G to H).

Further, we explored potential alterations in vimentin organization by analyzing its solubility via ultracentrifugation, thereby evaluating its integration into cytoskeletal networks. In *SNCA*^Iso^ midbrain neurons, vimentin was predominantly found in the insoluble fraction, whereas a notable shift toward the soluble fraction was observed in *SNCA*^Dupl^ midbrain neurons accompanied by increased truncated vimentin levels in both soluble and insoluble fractions (Fig. 7I). In summary, our analysis of vimentin in *SNCA*^Dupl^ midbrain neurons revealed significant effects of increased *SNCA* dosage on various aspects of vimentin biology, including elevated expression and truncation, reorganization from neuronal processes to cell bodies, and redistribution toward the soluble fraction.

### Vimentin interference reverses *SNCA* duplication–induced bTubIII and neurite disruptions

After identifying vimentin alterations specifically in response to *SNCA*^Dupl^, we explored whether changes in vimentin affect key aspects of *SNCA*^Dupl^-induced phenotypes. To address this, we interfered with vimentin organization using okadaic acid and withaferin A. Okadaic acid induces vimentin hyperphosphorylation and reorganization (*43*), while withaferin A directly binds to vimentin, influencing the integrity of the vimentin network (*44*, *45*).

**Fig. 8.**
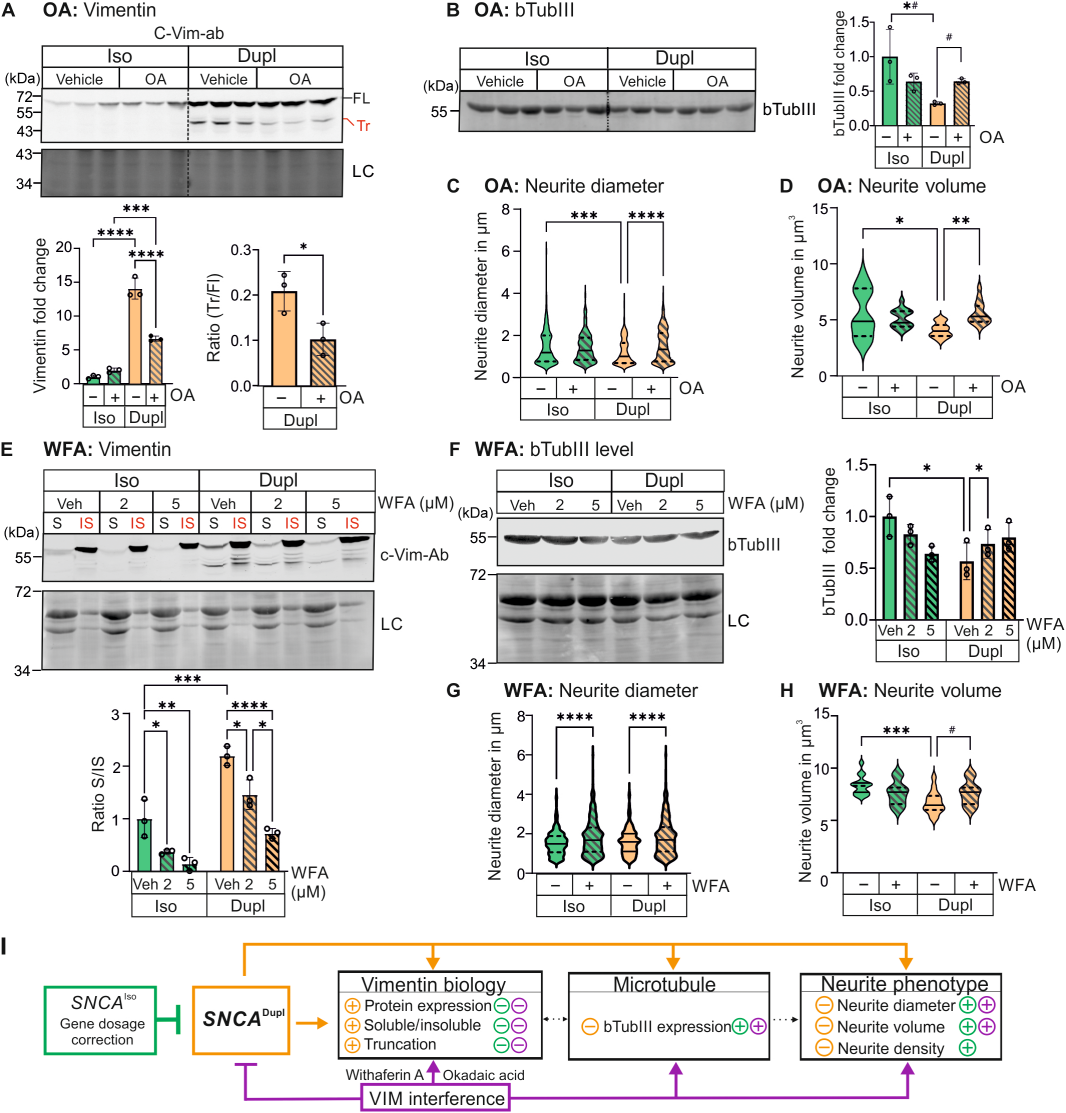
Effects of vimentin interference on vimentin, bTubIII, and neuritic phenotype. (**A**) WB analysis of vimentin in okadaic acid–treated neurons using the C-terminal vimentin antibody, showing reduced levels of total and truncated vimentin in *SNCA*^Dupl^ neurons upon treatment. (**B**) WB analysis of bTubIII, demonstrating reversed bTubIII levels in *SNCA*^Dupl^ neurons treated with okadaic acid. For (A) and (B), LC, Ponceau staining. Blots within the black frame were derived from the same membrane. Lanes from different parts of the same membrane are separated by dashed lines. (**C** and **D**) ICC analysis of neurite diameter and volume of bTubIII^+^ neurites. Okadaic acid treatment rescues the decrease in neurite diameter and volume observed in *SNCA*^Dupl^ neurons compared to *SNCA*^Iso^ neurons. (**E**) Solubility analysis of vimentin, showing a dose-dependent decrease in soluble vimentin levels in *SNCA*^Dupl^ neurons treated with 2 or 5 μM withaferin A (WFA), reversing the effect of *SNCA*^Dupl^ compared to *SNCA*^Iso^ neurons. (**F**) WB analysis of bTubIII levels, demonstrating a restoration of bTubIII levels in *SNCA*^Dupl^ neurons upon withaferin A treatment. For (E) and (F), LC, revert 520 Total Protein Stain. (**G** and **H**) ICC analysis of bTubIII^+^ neurites demonstrates that withaferin A treatment (2 μM) increases neurite diameter (G) and volume (H) in *SNCA*^Dupl^ neurons. (**I**) Schematic overview of the effects of vimentin interference on *SNCA*^Dupl^ phenotypes. Solid lines indicate findings revealed in this study, while dashed lines represent proposed interactions based on current data. For (C), (D), (G), and (H), neurite diameter was analyzed for 320 to 500 bTubIII^+^ neurites per group, and neurite volume was measured for 71-1142 bTubIII^+^ neurites per group. For all quantifications, *N* = 3 differentiation rounds.

Following okadaic acid treatment, we observed a significant reduction in vimentin levels and truncation in *SNCA*^Dupl^ neurons, effectively counteracting the key effects of *SNCA*^Dupl^ on vimentin when compared to *SNCA*^Iso^ neurons (Fig. 8A). In the present and our previous studies, *SNCA*^Dupl^ has been linked to impaired neurite phenotypes (*18*) and axonal transport dysfunction (*19*) in neurons derived from different patients with *SNCA*^Dupl^ compared to neurons of healthy donors, accompanied by a characteristic decrease in bTubIII levels (*18*, *46*). For this reason, we further investigated bTubIII levels via WB and observed a significant decrease in bTubIII levels in *SNCA*^Dupl^ neurons reversed by okadaic acid treatment (Fig. 8B). Moreover, okadaic acid significantly enhanced neurite diameter and volume in *SNCA*^Dupl^ midbrain neurons (Fig. 8, C and D), indicating a restoration of *SNCA*^Dupl^ neurite phenotypes.

The effects of targeting vimentin via withaferin A were analyzed by assessing vimentin levels, truncation, and solubility. Withaferin A did not induce a statistically significant decrease in total vimentin levels; however, a slight reduction was observed at the 5 μM application dose (fig. S8A). Notably, a decrease in truncated vimentin levels was observed, reaching statistical significance at a concentration of 5 μM withaferin A (fig. S8A). Intriguingly, withaferin A modulated the vimentin network in both *SNCA*^Iso^ and *SNCA*^Dupl^ neurons, as evidenced by a reduction in soluble vimentin (Fig. 8E). Notably, withaferin A treatment restored the increased soluble vimentin levels observed in *SNCA*^Dupl^ neurons. Similar to the effect of okadaic acid on bTubIII, withaferin A treatment resulted in an increase in bTubIII levels in *SNCA*^Dupl^ neurons (Fig. 8F). Neurite phenotype analysis revealed a significant increase in both neurite diameter (Fig. 8G) and neurite volume (Fig. 8H) in *SNCA*^Dupl^ neurons exposed to withaferin A. In addition, a restoration of neurite volume could be observed in treated *SNCA*^Dupl^ neurons compared to *SNCA*^Iso^ neurons (Fig. 8H).

In *SNCA*^Iso^ neurons, the effects of both vimentin-interfering compounds were less pronounced for most measured parameters (Fig. 8). In certain instances, such as bTubIII levels (Fig. 8, B and F) and neurite volume (Fig. 8, D and H), the effects even trended in the opposite direction compared to those observed in *SNCA*^Dupl^ neurons. These findings highlight the critical role of aSyn dosage in preserving neuronal integrity.

Given disturbed aSyn homeostasis observed in *SNCA*^Dupl^ neurons, we additionally analyzed aSyn following treatment of okadaic acid or withaferin A. WB analysis of aSyn levels revealed a significant increase in aSyn levels (~2-fold) in *SNCA*^Dupl^ neurons after okadaic acid treatment (fig. S8B), thus restoring aSyn levels, which tended to be reduced at the late neuronal differentiation stages compared to *SNCA*^Iso^ neurons (Fig. 2, A and B). However, aSyn levels remained unchanged following withaferin A treatment (fig. S8D), and neither compound restored the increased aSyn accumulation in neurites of *SNCA*^Dupl^ neurons (fig. S8, C and E). These results suggest that okadaic acid and withaferin A have distinct effects on aSyn levels but do not affect its spatial distribution within *SNCA*^Dupl^ neurons under our experimental conditions.

In summary, our study using two independent vimentin-interfering compounds highlights the potential of modulating vimentin network to restore *SNCA*^Dupl^-induced alterations in bTubIII levels and neurite phenotypes (Fig. 8I). These findings underscore the significance of vimentin in modulating the impact of increased *SNCA* dosage on cytoskeletal organization and ultimately neurite integrity.

## DISCUSSION

In this study, we identified specific effects of elevated *SNCA* gene dosage on dynamic aSyn changes during neuronal differentiation combined with alterations in the regulatory differentiation machinery and modifications in the cytoskeletal network. These molecular events resulted in a profound neurite phenotype at the cellular level. In particular, the data strongly underscore the critical interplay between neuronal aSyn and vimentin, both influencing neurite phenotypes.

Our findings provide notable insights into the effects of *SNCA* multiplication on aSyn dynamics during neuronal differentiation. Of particular interest is the initial rise in aSyn levels in differentiating *SNCA*^Dupl^ cells, followed by a pronounced decrease compared to control or *SNCA*^Iso^ neurons. This dynamic change is rarely reported in hiPSC-based studies, which predominantly focus on *SNCA* triplication and endpoint comparisons. However, a study by Fukusumi *et al.* (*47*) similarly observed a dynamic decline in aSyn levels during midbrain neuronal differentiation in *SNCA* triplication neurons, albeit using a growth factor–based protocol. The dynamic aSyn changes in *SNCA*^Dupl^ cells suggest a finely tuned regulation of aSyn homeostasis during neuronal differentiation. A plausible explanation for the reduced total aSyn levels in *SNCA*^Dupl^ neurons at advanced differentiation stages may involve the activation of compensatory mechanisms, such as the autophagy-lysosomal protein clearance pathway, in response to the initially elevated aSyn levels to degrade toxic forms of aSyn. Despite the overall reduction in total aSyn levels, we detected a significant increase in aSyn aggregation in *SNCA*^Dupl^ neurons, along with a pronounced neuritic accumulation of aSyn in *SNCA*^Dupl^ neurons, especially evident when using an aSyn aggregate–sensitive antibody. These findings indicate a shift in aSyn homeostasis toward aggregation and a spatial redistribution of aggregated aSyn to neurites, correlating with the neurite deficits in these neurons. Elevated aSyn accumulation within neurites can disrupt crucial cytoskeleton-dependent transport processes between the cell body and nerve terminals, leading to neurite dysfunction, as evidenced in prior research (*10*, *11*, *48*), including our own (*19*). Given that dopaminergic neurons are the predominant neuronal subtype in our model, they may likely be the primary contributor to the distinct pattern of intraneuronal aSyn dynamics we observed. This pattern may contrast with findings in the limited postmortem brain studies of *SNCA* multiplication patients, where increased soluble aSyn protein levels have been reported (*49*). The differences may reflect the complexity of cell populations in postmortem tissue, which contribute to a more heterogeneous landscape of aSyn regulation.

Neurite deficits may be a result of disturbed neuronal differentiation pathways in *SNCA*^Dupl^, as evidenced by comprehensive transcriptome and proteome analyses in our study. Moreover, our ATAC-seq data, showing *SNCA*^Dupl^-mediated changes in the chromatin landscape, support a regulatory role of aSyn at the transcriptional levels. Particularly noteworthy was the pronounced down-regulation of PAX6 expression in *SNCA*^Dupl^ NPCs and midbrain neurons compared to their respective *SNCA*^Iso^ counterparts, suggesting an intricate interplay between aSyn and PAX6. Tight regulation of PAX6 is crucial for balancing cell proliferation, neuronal differentiation, and proper brain patterning (*50*). Specifically, in dopaminergic neurons, PAX6 deficiency has been linked to abnormal neuronal localization and impaired axonal pathfinding in murine brains (*51*). These effects may be associated with disturbances in the ECM organization, which play a key role in the axonal guidance environment. Furthermore, a reduction in PAX6 immunoreactive cells has been documented in the postmortem brain of patients with PD (*52*). Our findings highlight the impact of elevated *SNCA* dosage, which triggers reduced PAX6 expression, alongside alterations in ECM organization and neurite deficits. However, the exact regulatory mechanisms through which aSyn influences PAX6 remains unclear. Considering the nuclear localization of aSyn (*20*, *53*) and accumulating evidence for its role as a transcriptional modulator (*54*), nuclear aSyn may participate in the transcriptional regulation of PAX6 expression (i) indirectly via modification of histones (*55*) or interaction with collaborating transcription factors or (ii) directly via binding to the *PAX6* gene, thus acting as a transcription factor (*56*). Exploring whether and how aSyn regulates transcription and in particular PAX6 expression could be further investigated in future studies using genomics approaches, such as transcription factor chromatin immunoprecipitation followed by sequencing.

hiPSC cells generated from individuals with *SNCA* multiplication exhibit variations in the length of the multiplication stretch (*57*). Some phenotypes described in this study, such as disrupted aSyn homeostasis, altered bTubIII expression, and neurite abnormalities, were consistently observed in *SNCA*^Dupl^ neurons. These findings were replicated in comparisons with both neurons from healthy donors without any *SNCA* mutations [in this and our previous study (*18*)] and isogenic control neurons (in this study). These results suggest that the effects observed are primarily attributable to *SNCA*^Dupl^. However, the contribution of other genes flanking the *SNCA* gene within the duplicated genomic region cannot be excluded. One such neighboring gene is *GPRIN3*, directly located at the 3′ site of *SNCA*. It was unexpectedly identified in our study using unbiased multi-omics approaches as one of the most down-regulated features in *SNCA*^Dupl^ neurons at both the mRNA and protein levels. While the specific function of the GPRIN3 protein, particularly in the context of PD, remains undefined, a study by Karadurmus *et al.* (*30*), characterizing a *GPRIN3* knockout mouse model, demonstrated the role of GPRIN3 protein as a mediator of dopamine receptor D2R in the striatum. This finding suggests the GPRIN3 protein as a functional partner of the midbrain dopaminergic pathway. Our finding of reduced *GPRIN3* gene expression in *SNCA*^Dupl^ midbrain neurons could be explained by regulatory interaction of *SNCA* with *GPRIN3*, both possibly sharing common regulatory genomic features. The potential contribution of neighboring genes in the *SNCA* multiplication region is largely unexplored and should be considered in future studies. Further functional investigations are necessary to dissect the individual effects and elucidate interplay of *SNCA* and neighboring genes affected by multiplication. These studies will provide a more comprehensive understanding of the mechanisms underlying *SNCA* multiplication–related phenotypes.

Our previous studies comparing midbrain neurons from different *SNCA*^Dupl^ cases and control donors revealed a direct interaction between aSyn and microtubule elements (*18*). The microtubule network, composed of α- and β-tubulin subunits, is a key component of the cytoskeleton and plays a pivotal role in neurite organization and development. Notably, we observed a significant reduction in bTubIII across all investigated *SNCA*^Dupl^ cases (*18*, *46*), alongside increased coaggregation and co-redistribution of aSyn and bTubIII (*18*). In our present study comparing *SNCA*^Dupl^ and *SNCA*^Iso^ midbrain neurons, we highlight the specific effect of elevated *SNCA* dosage on bTubIII reduction, linked to a massive decrease in neurite network formation and parameters associated with neurite development such as neurite diameter and volume.

An important finding of this study is the identification of the interaction between aSyn homeostasis and the vimentin network. Vimentin, an essential intermediate filament protein, represents another crucial component of the cytoskeleton in addition to microtubule. While previous research predominantly focused on its role in astrocytes, particularly in astrogliosis (*58*), recent studies have highlighted its significance in neuronal differentiation, axon outgrowth, and regenerative processes in neurons and neurites (*59*). Beyond its role as a cellular structural organizer, vimentin has been demonstrated to inhibit the long noncoding RNA Gas5, which is expressed age-dependently and suppresses axonal regeneration (*60*). In the context of neurodegeneration, neuronal vimentin has been observed in the cytoplasm and dendrites of neurons in the cortex, hippocampus, and cerebellum of patients with Alzheimer’s disease, regions known to exhibit intra- and extracellular amyloid-β (1–42) (Abeta42) deposition. Colocalization of neuronal vimentin and Abeta42 is prevalent in these brain regions (*61*).

In this study, we shed light on the multifaceted impact of increased *SNCA* dosage on vimentin biology within neurons. This encompasses a notable increase in vimentin levels, enhanced truncation (especially evident in differentiated neurons), a shift from insoluble to soluble fractions (indicating its release from the cytoskeletal network), and its redistribution from neurites to the soma. Although the relevant data are limited, several studies have suggested a regulatory role of aSyn in vimentin: A significant decrease in vimentin levels upon depletion of aSyn expression was demonstrated in *SNCA* knockout melanoma cells; conversely, in SH-SY5Y cells, overexpression of aSyn led to a drastic increase in vimentin levels (*62*). Another study, which induced aggresomal structures resembling LB via Parkin overexpression, confirmed the presence of both aSyn and vimentin within these structures (*63*). Vimentin was reported to coordinate the transport of proteasome to the aggresome, thereby contributing to proteostasis (*64*). Regarding vimentin truncation, limited proteolysis of vimentin from both the N and C termini has been documented in several studies, demonstrating that vimentin truncation significantly increases its solubility, thereby interrupting its assembly (*65*–*67*). Our data obtained from *SNCA*^Dupl^ cell model further support this notion. Intriguingly, both vimentin levels and truncation were markedly elevated in mouse models of PD and MSA overexpressing human aSyn. Similar patterns were also observed in postmortem putamen from patients with sporadic PD. Proteases, such as caspases and calpains (*65*, *66*), have been shown to cleave vimentin, producing various truncated vimentin forms. Notably, studies on postmortem brains and PD animal models have reported elevated calpain levels and activity, as well as the protective effects of calpain inhibition (*68*–*70*). Further research is needed to identify the precise cleavage sites involved in aSyn-mediated vimentin truncation. Overcoming the technical challenges in isolating specific truncated forms and dissecting their functions in biological models will be crucial. The regulatory mechanisms driving aSyn-mediated vimentin truncation, including the role of proteases, require further investigation.

While elucidating the effect of increased *SNCA* dosage on vimentin organization, we also highlighted the impact of vimentin interference on key phenotypes observed in *SNCA*^Dupl^ midbrain neurons. Both okadaic acid and withaferin A, despite different mechanisms of action, reversed vimentin-related phenotypes. Okadaic acid reduced vimentin levels, withaferin A restored the proportion of soluble vimentin, and both vimentin parameters were altered in *SNCA*^Dupl^ neurons. In addition, both compounds corrected deficits in bTubIII levels and neurite structure. These significant findings suggest that *SNCA*^Dupl^-mediated vimentin alterations precede the microtubule- and neurite-associated phenotypes in midbrain neurons. Considering the emerging evidence in regard to the neuroprotective effects of withaferin A in PD-related cell and animal models (*71*, *72*), our findings highlight the intervention potential of targeting the vimentin pathway to protect against aSyn-mediated neurite degeneration, a very early cellular event in neurodegeneration. To validate this potential, further precise and comprehensive studies using more targeted interference of vimentin, such as genetic modification, are required. Moreover, further validation of compounds like withaferin A is necessary to explore their effects on the interplay between aSyn, vimentin, as well as neurite structure and function in different neural cell types using both cell-based and in vivo models, to assess their clinical potential.

The vimentin phenotypes demonstrated in our studies are either less pronounced or rarely documented in other hiPSC-based neuronal models derived from *SNCA* triplication. This discrepancy may partly be influenced by variations in *SNCA* copy number. Clinical studies have provided evidence that different *SNCA* copy numbers in multiplication patients influence the penetrance of symptoms as well as disease onset and progression (*73*–*75*). In addition, various neighboring multiplication loci of *SNCA* may also contribute to these differences, underscoring the need for consideration in future studies.

In summary, our present study highlights the specific impact of *SNCA*^Dupl^ on neuronal differentiation and neurite development through cytoskeletal disruption. *SNCA* dosage correction of aSyn to a normal level is able to restore these effects. Our findings further emphasize the modulatory potential of targeting the vimentin network.

## MATERIALS AND METHODS

### Experimental design with human-derived materials

#### Human iPSCs

hiPSCs were derived from dermal fibroblasts obtained through skin biopsies from a patient with PD and healthy donors. Skin biopsies were conducted at the Movement Disorder Clinic, Department of Molecular Neurology, University Hospital Erlangen, following written informed consent obtained from voluntary donors. All experiments involving hiPSC-derived cells were conducted in accordance with Institutional Review Board approval (Nr. 259_17B) guidelines.

Skin biopsy specimens were collected from a female patient with PD carrying *SNCA* duplication (*SNCA*^Dupl^) (*3; table S1), as well as from two healthy individuals without history of neurological disease (*1 and *2; table S1). The patient with PD, who was 44 years old at the time of biopsy, began experiencing disease symptoms at the age of 39. The presence of *SNCA*^Dupl^ was confirmed using next-generation sequencing, which revealed a heterozygous duplication of a DNA region on chromosome 4q22.1. The minimal estimated duplicated region spans from 88,007,635 to 91,301,913 (3.59 Mb), while the maximal estimated duplicated region extends from 87,987,426 to 92,303,966 (4.32 Mb). The healthy donors, a male (*1) and a female (*2), were respectively 69 and 42 years old at the time of biopsy. Two PD-hiPSC clones [UKERi7GG-S1-004 (hiPSC clone *SNCA*^Dupl^#1) and UKERi7GG–S1-005 (hiPSC clone *SNCA*^Dupl^#2], derived from the patient with PD *SNCA*^Dupl^ (*3) and three control hiPSC lines (UKERiG3G-R1-039 from donor *1 (hiPSC clone Ctrl#1); UKERi1JF-R1-011 (hiPSC clone Ctrl#2) and UKERi1JF-R1-018 (hiPSC clone Ctrl#3) from donor *2 were used in this study (table S1).

CRISPR-Cas9 gene editing was conducted by using the PD *SNCA*^Dupl^#1 hiPSC line, generating the hiPSC line *SNCA*^Iso^. Details regarding the skin biopsy donors and the hiPSC lines generated for this study are provided in table S1 and in our previous publication (*24*). Unless specifically indicated in the figure legends, hiPSC lines Ctrl#3, *SNCA*^Dupl^#2, and *SNCA*^Iso^ were used in analyses.

#### Human postmortem tissue

Human postmortem samples of five patients with PD and five age- and gender-matched controls (table S2) were obtained from the Netherlands Brain Bank, Netherlands Institute for Neuroscience, Amsterdam (open access: http://brainbank.nl). PD cases were clinically diagnosed by the presence of motor symptoms (bradykinesia, rigidity, and tremor) and neuropathologically confirmed by the occurrence of LB pathology. In this study, all PD cases exhibited neocortical LB, according to Braak Lewy stages 5 and 6 (*76*).

### Generation of an isogenic control hiPSC line through heterozygous knockout of *SNCA* in a *SNCA*^Dupl^ hiPSC line

For the design of single guide RNAs (sgRNAs) targeting *SNCA* gene, the web tool CRISPOR was used (http://crispor.tefor.net/). Two sgRNAs were selected flanking the whole exon 2 of *SNCA* (table S3). Synthetic sgRNAs and recombinant Cas9 were both purchased from Synthego, CA, USA. The subsequent *SNCA* gene editing in the *SNCA*^Dupl^ hiPSC line was performed as described previously (*24*) and summarized in fig. S1A.

Specifically, hiPSCs were cultivated in mTeSR on a six-well plate precoated with GeltrexTM (Thermo Fisher Scientific, concentration according to the manufacturer’s recommendation) in mTeSR Plus (STEMCELL Technologies) with 1% penicillin-streptomycin at 37°C and 5% CO_2_. For CRISPR-Cas9, hiPSCs were dissociated using Accutase (Sigma-Aldrich) and nucleofected with the synthetic sgRNAs. Before transfection, 100 μM sgRNAs were mixed with 20 μM of recombinant Cas9 in a ratio of 7.5:1 and incubated for 10 min at room temperature (RT) for in vitro assembly. hiPSCs were replated on six-well plates for 48 hours before single-cell isolation. Single cells were isolated via flow cytometry on 96-well plates in mTeSR Plus containing CloneR (STEMCELL Technologies). After reaching confluency of 80 to 100%, each clone was split 1:2 into two 48-well plates for genotyping and downstream validation analyses. DNA of clones was isolated using QuickExtract (Lucigen) according to the manufacturer’s protocol, and DNA was amplified with the Q5 High-Fidelity DNA Polymerase (New England Biolabs) using *SNCA*-specific primers (table S3) and separated by agarose gel electrophoresis (Fig. 1B). Modified DNAs of candidate clones were isolated from agarose gel using QIAquick PCR Purification Kit (Qiagen) and validated by sequencing (LGC Genomics, Berlin; fig. S1B). Obtained sequences were prescreened for genetic insertion and deletion (indel) using the Inference of CRISPR Edits tool provided by Synthego (https://ice.synthego.com/#/) (fig. S1C). The candidate clone showing an indel frequency between ~20 and 40% was harvested and verified for modification by sequencing (LGC Genomics, Berlin, Germany). Ultimately, modifications were confirmed by expression analysis via WB and RT-qPCR. In addition, off-target analysis was performed amplifying the five most homologous genomic sequences provided by the CRISPOR web tool (fig. S1E).

### Differentiation of midbrain neurons from hiPSC

Midbrain neurons were generated by small molecule–based induction of hiPSCs according to Reinhardt *et al.* (*25*) and previously published studies by our group introducing minor modifications (*18*, *46*). For the generation of NPCs, neural induction medium (Nid0), including mTeSR Plus supplemented with 1 μM LDN, 10 μM SB-431542, 3 μM Chir, 0.5 μM purmorphamine (PurMA), and 10 μM Y-27632 2HCL (ROCK inhibitor) was initially used. HiPSCs were dissociated using collagenase IV and resuspended in this Nid0 medium on Ultra-Low Adhesion Surface Plates (Corning). After 24 hours, the Nid0 medium was replaced by medium without ROCK inhibitor (Nid1). On day 3, Nid1 was replaced by N2B27 medium, consisting of 50% Dulbecco’s modified Eagle’s medium/F12 Glutamax, 50% Neurobasal medium, 1:200 N2, 1:100 B27, 1:100 penicillin-streptomycin supplemented with all factors mentioned above (Nid2). On day 4, LDN was withdrawn from Nid2 and supplemented with 150 μM ascorbic acid (smNPC medium). On day 6, the generated embryoid bodies were dissociated using a P1000 pipette and transferred to 12-well plates in a 1:4 ratio in smNPC medium supplemented with 5 μM ROCK inhibitor. After 24 hours, ROCK inhibitor was withdrawn and cells were cultivated until 80 to 100% confluency. For NPC passaging, the cells were detached with Accutase (Sigma-Aldrich) and split in 1:3 to 1:5 ratios on 12-well plates coated with Geltrex according to the manufacturer’s instruction. NPCs were frozen in Knockout SR, supplemented with 10% dimethyl sulfoxide (DMSO), and stored at −150°C or immediately used for differentiation into midbrain neurons.

For midbrain differentiation, NPCs of more than eight passages were cultivated on 12-well plates in smNPC medium until 80 to 100% confluency. Afterward, the smNPC medium was replaced by N2B27 medium containing FGF-8 (100 ng/ml), 1 μM PurMA, and 200 μM ascorbic acid (differentiation medium). The cells were cultivated in differentiation medium until day 8 of differentiation, and medium was renewed every other day. On day 8 of neuronal differentiation, differentiation medium was replaced by medium containing brain-derived neurotrophic factor (10 ng/ml), glial cell line–derived neurotrophic factor (10 ng/ml), transforming growth factor–β3 (1 ng/ml), 500 μM cyclic adenosine monophosphate, 0.5 μM PurMA, and 200 μM ascorbic acid (maturation medium). On day 9, 0.75 × 10^6^ cells were transferred to 12-well plates in maturation medium supplemented with 10 μM ROCK inhibitor and removed after additional 24 hours. On day 11, PurMA was withdrawn from maturation medium, and the cells were cultivated in this medium until day 24 of maturation, replacing maturation medium every other day. Unless otherwise specified, midbrain neurons differentiated for 24 days were used for the analyses.

### Reverse transcription quantitative PCR

For RT-qPCR, cell pellets were homogenized in RLT buffer containing 1% β-mercaptoethanol (74136, Qiagen) and RNA isolated according to the manufacturer’s protocol. RNA concentration was determined via Nano-Drop (PeqLab). Complementary DNA was generated using the GoScript Reverse Transcription System (A5004, Promega). For quantification of transcript levels, qPCR was performed on a LightCycler 480 (Roche) using the SsoFast EvaGreen Supermix (1725205, Bio-Rad Laboratories) with specific primers. The program contained the following five steps: (i) enzyme activation for 30 s at 95°C; (ii) denaturation for 5 s at 95°C; (iii) annealing for 20 s at 64° to 66°C depending on optimal temperature for primers; (iv) acquisition for 20 s at 73°C; (v) melt curve with continuous temperature elevation from 65°C to 95°C. While steps (i) and (v) were performed only once at the beginning and the end of the program, respectively, 55 cycles of steps (ii), (iii), and (iv) were conducted. For qPCR analysis, each sample was measured in duplicates. Gene expression levels were determined using LightCycler480 Software (Roche) and normalized to housekeeping genes 18*S* and glyceraldehyde-3-phosphate dehydrogenase. All primers used are given in table S3.

### WB analyses

For WB analysis, the cells were harvested in cold phosphate-buffered saline (PBS) and centrifuged at 300*g* for 5 min at 4°C. Unless stated otherwise, the pellet was resuspended in radioimmunoprecipitation assay (RIPA) lysis buffer [50 mM tris/HCl (pH 8.0), 150 mM NaCl, 5 mM EDTA, 1% NP-40, 0.5% sodium deoxycholate, and 0.1% SDS], followed by three freeze and thaw cycles at −80°C and +37°C and incubation on ice for 30 min. For the preparation of human brain tissue, the samples were first homogenized in homogenization buffer [50 mM tris/HCl and 150 mM NaCl (pH 8)] using a Braun Potter S Homogenizer (Sartorius AG), then mixed with 4× RIPA in a ratio of 1:3, and incubated on ice for 30 min. After centrifugation at 10,000*g* for 20 min at 4°C, the protein concentration of the supernatant was determined using the Pierce bicinchoninic acid assay (BCA, 23225, Thermo Fisher Scientific). Afterward, 20 to 30 μg of total protein was either mixed with 4× LDS and 10× reducing agent (all from Thermo Fisher Scientific) or with 5× Laemmli buffer [0.3 M tris/HCl (pH 6.8), 50% glycerol, 1% SDS, 0.05% bromophenol blue, and 5% 2-mercaptoethanol], followed by incubation at 70°C for 10 min (for samples in LDS buffer) or 99°C for 10 min (for samples in Laemmli buffer). SDS-PAGE was performed using MES buffer on NuPAGE 4 to 12% bis-tris protein gels (Thermo Fisher Scientific) or using tris/glycine buffer with selfcast 12% acrylamide gels at 120 V for 1 hour and 30 min. For detection of vimentin truncation, Laemmli buffer and 12% acrylamide gels were used. Protein was transferred to a polyvinylidene fluoride (PVDF-FL) membrane in a Wet/Tank Blotting System (Bio-Rad) at 90 V for 1.5 hours. Membranes were fixed using 4% paraformaldehyde (PFA) before blocking in 1% bovine serum albumin (BSA) in TBS-T for 1 hour at RT. Primary antibody incubation was performed at 4°C overnight followed by incubation with fluorophore-coupled secondary antibodies for 1.5 hours. All primary and secondary antibodies and their working dilutions are listed in table S4. Before imaging, the membranes were dried in the dark to improve signal contrast. Protein loading control (LC in WB images) was carried out by staining the gel with Coomassie Brilliant Blue G 250 (Bio-Rad), or the PVDF-FL membrane was stained with either Ponceau S (Sigma-Aldrich) or Revert 520 Total Protein Stain (Li-Cor Bioscience) according to the manufacturer’s instruction. Membranes were imaged either using a Fusion FX7 system (Peqlab) or an Odyssey M (Li-Cor Biosciences) imaging system.

### Solubility assay

For assessment of protein solubility, the cells were homogenized in 50 mM tris/HCl (pH 7.4), 175 mM NaCl, 5 mM EDTA, and 1% Triton X-100 using a Braun Potter S Homogenizer (Sartorius AG). Protein concentration was determined using the BCA assay and adjusted to 1 μg/μl, after which 100 μl of the protein homogenate was processed further. The homogenate was centrifuged at 100,000*g* for 60 min at 4°C using the Sorvall WX+ ultracentrifuge (Thermo Fisher Scientific). The pellet was resuspended in half the volume of the supernatant using a buffer containing 0.5 M urea and 5% SDS. Both fractions were mixed with 5× Laemmli buffer. After boiling at 99°C for 10 min, the samples were loaded on 12% SDS-PAGE gels for electrophoresis. WB and immunostaining were carried out as described above.

### Enzyme-linked immunosorbent assay

Levels of total and aggregated aSyn were analyzed using the LEGEND MAX Human a-Synuclein ELISA kit (BioLegend, catalog no. 448607) and LEGEND MAX a-Synuclein Aggregate ELISA Kit (BioLegend, catalog no. 449407) following the manufacturer’s instructions. Specifically, cells were harvested in PBS and resuspended in a lysis buffer, containing 20 mM Hepes, 150 mM NaCl, 1 mM EDTA, 1.5 mM MgCl_2_, 10% glycerol, and 1% Triton X-100. After three freeze-thaw cycles (−80° and 37°C), the cell lysate was incubated on ice for 30 min and centrifuged at 10,000*g* for 10 min. The resulting supernatant was used to measure total and aggregated aSyn using 0.1 and 20 μg of total protein, respectively.

### Immunocytochemistry

For ICC, cells were initially fixed with 2% PFA for 10 min, followed by a subsequent fixation step using 4% PFA for an additional 10 min. Following cell fixation, PBS was used to rinse the cells three times at RT. To minimize nonspecific antibody binding, the cells were treated with fish skin gelatin buffer (FSGB: tris-buffered saline with 0.4% cold water fish skin gelatin in water, 1% BSA, and 0.1% Triton X-100) for 30 min at RT. Primary antibodies, diluted in FSGB, were then applied and incubated for 1 hour at RT. After primary antibody incubation, the cells were rinsed three times with PBS at RT, followed by incubation with the appropriate secondary antibody for 1 hour at RT. Nuclei were counterstained with 4′,6-diamidino-2-phenylindole (0.1 μg/ml) for 5 min at RT. Subsequently, the cells were washed three more times with PBS and mounted using ProLong solution before imaging. All antibodies used for ICC are listed in table S4. Imaging was performed using the fluorescence Observer microscope and a Zeiss LSM 780 confocal laser scanning microscope (Carl Zeiss Microscopy GmbH, Jena, Germany).

### Analysis of neuronal and neurite phenotypes

The distribution of aSyn in neurites and neuronal somata was assessed using Zen 2013 software. Each neuron was examined by randomly selecting one or two primary neurites. Cytosolic and neuritic regions were delineated, and the intensity of aSyn signals was quantified in both compartments.

For analyzing neurite parameters, each neuron was examined by randomly selecting one or two primary neurites. Neurite diameter and volume were quantified using IMARIS (Bitplane, Zurich, Switzerland). The filament tracing feature of the software was used to automatically trace neurites within a randomly chosen image area (fig. S3A). Neurite diameter analysis focused solely on primary neurites before the first branching point, whereas neurite volume determination involved neurites spanning from the first to the fifth branching point.

Neurite density was assessed using fluorescent images of neurons stained for bTubIII, recorded with a 10× objective on a Zeiss LSM 780 microscope and analyzed using ImageJ software (*77*). Five regions of interest per image were unbiasedly selected and processed with consistent parameters, including thresholding (15 pixels) to binarize the image and background subtraction (10 pixels). Neurites were skeletonized, and the resulting two-dimensional (2D)/3D skeletons were analyzed to calculate the number of skeletons per area (in pixels^2^) as a measure of neurite density.

Immunofluorescence staining of vimentin and bTubIII was imaged using the Zeiss LSM microscope. Quantification of vimentin immunoreactive neurites was performed using the Fiji Plugin of the ImageJ software. For this, the focus was set on neuronal cell accumulations showing neuritic outgrowth. Absolute number of neurites was assessed counting the bTubIII^+^ neurites using the cell counter plugin of ImageJ. For calculation of the proportion of the Vim^+^/bTubIII^+^ neurites, the number of Vim^+^ neurites was assessed and normalized to the absolute number of bTubIII^+^ neurites.

### MS-based proteomics

#### Sample preparation and mass spectrometric measurements

All samples were prepared in 96-well plate format using the optimized SP3 protocol according to Hughes *et al.* (*78*). Specifically, cell pellets were lysed in 1% NP-40 and 0.2% (w/v) SDS in 20 mM Hepes (pH 7.5). Protein concentration in lysates was determined using BCA (Thermo Fisher Scientific, #23225). Protein amount was adjusted to 10 μg in a total volume of 40 μl of lysis buffer. The protein was loaded onto a 1:1 mixture of hydrophilic and hydrophobic carboxylate–coated magnetic beads (Cytiva, #45152105050250 and #65152105050250, 10 μl each) prewashed three times with 100 μl of MS-grade H_2_O (Honeywell, #15665350). The magnetic beads with protein samples were mixed at 850 rpm for 1 min at RT. To initiate the binding, 60 μl of absolute EtOH was added, and the mixture was incubated at RT for 5 min at 850 rpm. Subsequently, the beads were washed three times with 80% (v/v) EtOH, with incubation at RT for 1 min and 850 rpm between each wash. After the last wash, the beads were resuspended in 50 μl of 100 mM ammonium acetate buffer (ABC, Sigma-Aldrich, #09689). The wash steps and ABC buffer addition were performed by a liquid handling robot (Hamilton Microlab Prep). The on-beads digestion was performed with trypsin (Promega, V5113, 1 μg) overnight at 37°C and 850 rpm. The resulting peptide mixture was eluted from the magnetic beads into a new 1.5-ml tube. The magnetic beads were washed with 50 and 30 μl of 1% (v/v) formic acid (TCI, # F0654) and incubated at 40°C at 850 rpm for 5 min. The fractions were added to the first elution fraction. The combined fractions were further purified from remaining magnetic beads. MS measurements were performed on an Orbitrap Eclipse Tribrid Mass Spectrometer (Thermo Fisher Scientific) coupled to an UltiMate 3000 Nano-HPLC (Thermo Fisher Scientific) via a nanospray Flex ion source (Thermo Fisher Scientific) equipped with column oven (Sonation) and FAIMS interface (Thermo Fisher Scientific). Peptides were loaded on an Acclaim PepMap 100 μ-precolumn cartridge (5 μm, 100 Å, 300 μm ID by 5 mm, Thermo Fisher Scientific) and separated at 40°C on a PicoTip emitter (noncoated, 15 cm, 75 μm ID, 8 μm tip, New Objective) that was in-house packed with Reprosil-Pur 120 C18-AQ material (1.9 μm, 150 Å, Dr. A. Maisch GmbH). Buffer composition: buffer A consists of MS-grade H_2_O supplemented with 0.1% formic acid (FA); buffer B consists of acetonitrile supplemented with 0.1% FA. The 105-min LC gradient from 4 to 35.2% buffer B was used. The flow rate was 0.3 μl/min.

#### Data-independent acquisition

The DIC duty cycle comprised one MS1 scan followed by 30 MS2 scans. The MS2 scans had an isolation window of 4 mass/charge ratio (*m/z*) range, which overlapped with an adjacent window at the 2 *m/z* range. MS1 scan was conducted with Orbitrap at 60,000 resolution power and a scan range of 200 to 1800 *m/z* with an adjusted radio frequency (RF) lens at 30%. MS2 scans were conducted with Orbitrap at 30,000 resolution power, and RF lens was set to 30%. The precursor mass window was restricted to a 500 to 740 *m/z* range. HCD fragmentation was enabled as an activation method with a fixed collision energy of 35%. FAIMS was performed with one CV at −45 V for both MS1 and MS2 scans during the duty cycle.

#### Computational evaluation of DIA raw files

Raw files were converted in the first step with “MSConvertGUI” as a part of the “ProteoWizard” software package (http://www.proteowizard.org/download.html) to an output mzML format applying the “peakPicking” filter with “vendor msLevel = 1,” and the “Demultiplex” filter with parameters “Overlap Only” and “mass error” set to 10 ppm. Standalone DIA-NN software under version 1.8.1 was used for protein identification and quantification. First, a spectral library was predicted in silico by the software’s deep learning-based spectra, RTs, and IMs prediction using UniProt database for Homo sapiens (taxon identifier: 9606; containing canonical and isoforms, decoys, and common contaminants). DIA-NN search settings are as follows: FASTA digest for library-free search/library generation option was enabled, together with a match between runs option and precursor FDR level set at 1%. Library generation was set to smart profiling, Quantification strategy - Robust LC. The mass accuracy and the scan window were set to 0 to allow the software to identify optimal conditions. The precursor *m/z* range was changed to 500 to 740 *m/z* to fit the measuring parameters.

#### Proteome analysis

Perseus (2.0.9.0) was used to log_2_ transform LFQ intensities, replace missing values from normal distribution, and construct the volcano plots (*79*).

### RNA-seq library preparation

RNA-seq libraries were prepared as previously described (*80*). Approximately ~200,000 iPSC-derived neurons were collected in TRIzol. Total RNA was extracted from cells using the Direct-zol RNA MicroPrep Kit (Zymo Research R2052) and stored at −80°C until RNA-seq library preparation. mRNAs were enriched by incubation with Oligo d(T) magnetic beads (NEB, S1419S) in 1× DTBB buffer [20 mM tris-HCl (pH 7.5), 1 M LiCl, 2 mM EDTA, 1% lithium dodecyl sulfate, and 0.1% Triton X-100] at 65°C for 2 min and were incubated at RT while rotating for 10 min. The beads were washed 1× with RNA wash buffer 1 [10 mM tris-HCl (pH 7.5), 0.15 M LiCl, 1 mM EDTA, 0.1% lithium dodecyl sulfate, and 0.1% Triton X-100] and 1× with RNA wash buffer 3 [10 mM tris-HCl (pH 7.5), 0.15 M NaCl, and 1 mM EDTA] before elution in RNA elution buffer [10 mM tris-HCl (pH 7.5) and 1 mM EDTA] at 80°C for 2 min. PolyA selection was performed a second time, and samples were washed 1× with wash buffer 1, 1× with wash buffer 3, and 1× with 1× SuperScript III first-strand buffer. Beads were resuspended in 10 μl 2× SuperScript III buffer plus 10 mM dithiothreitol (DTT), and RNA was fragmented at 94°C for 9 min and immediately chilled on ice before the next step. For first-strand synthesis, 10 μl of fragmented mRNA, 0.5 μl of random primers (50 μM) (Thermo Fisher Scientific), 0.5 μl of SUPERase-In (Ambion), 1 μl of deoxynucleotide triphosphates (dNTPs, 10 mM), and 0.5 μl of oligo(dT)20 Primer (Thermo Fisher Scientific) were heated for 50°C for 1 min. At the end of incubation, 5.8 μl of water, 1 μl of DTT (100 mM), 0.1 μl of actinomycin D (2 μg/μl), 0.2 μl of 1% Tween-20 (Sigma-Aldrich), and 0.5 μl of SuperScript III (Thermo Fisher Scientific) were added and incubated in a PCR machine using the following conditions: 25°C for 10 min and 50°C for 50 min. The product was then immediately purified with RNAClean XP beads (Beckman Coulter) according to the manufacturer’s instruction and eluted with 10 μl of nuclease-free water. The RNA/cDNA double-stranded hybrid was then added to 1.5 μl of Blue Buffer (Enzymatics), 1.0 μl dNTP mix with deoxyuridine triphosphate (dUTP) (10 mM each deoxyadenosine triphosphate, deoxycytidine triphosphate, deoxyguanosine triphosphate, and dUTP), 0.1 μl of dUTP (100 mM), 0.2 μl ribonuclease H (5 U/μl), 1.05 μl of water, 1 μl of DNA polymerase I (Enzymatics), and 0.15 μl of 1% Tween-20. The mixture was incubated at 16°C overnight. The following day, the dUTP-marked double-stranded DNA (dsDNA) was purified using 28 μl of SpeedBeads (GE Healthcare), diluted with 20% polyethylene glycol, molecular weight 8000 (PEG8000) and 2.5 M NaCl to a final concentration of 13% PEG, and eluted with 40 μl of elution buffer (DNA elution buffer from Zymo ChIP Clean and Concentrator Kit). The purified dsDNA underwent end repair by blunting, A-tailing, and adaptor ligation as previously described (*81*) using barcoded adapters (NextFlex, Bioo Scientific). Libraries were PCR amplified for 12 cycles, size selected for 200 to 500 bp, quantified using a Qubit dsDNA HS Assay Kit (Thermo Fisher Scientific), and sequenced on a NovaSeq 6000 for 51 cycles according to the manufacturer’s instructions.

### ATAC-seq library preparation

ATAC-seq libraries were prepared as follows: Approximately 1 × 10^6^ iPSC-derived neurons were resuspended in 47.5 μl of lysis buffer [10 mM tris-HCl (pH 7.5), 10 mM NaCl, 3 mM MgCl_2_, 0.1% IGEPAL, and CA-630 in water] at RT. After the addition of 2.5 μl of DNA enzyme I (Illumina), the cells were incubated at 37°C for 30 min. DNA was purified with Zymo ChIP DNA concentrator columns (Zymo Research), eluted with 11 μl of elution buffer, and amplified using NEBNext High-fidelity 2× PCR MasterMix (New England BioLabs) with the Nextera primer Ad1 (1.25 μM) and a unique Ad2.n barcoding primer (1.25 μM) for 8 to 10 cycles. The resulting libraries were size selected by gel excision to 165 to 400 bp, purified, and single-end sequenced using a NovaSeq 6000 (Illumina) sequencer for 51 cycles according to the manufacturer’s instructions.

### RNA-seq analysis

FASTQ files from RNA-seq experiments were mapped to hg38 using STAR (*82*) with default parameters. After mapping, tag directories were created using the HOMER command makeTagDirectory. The gene expression raw counts were quantified by HOMER’s (*81*) analyzeRepeats command with the option “-condenseGenes -count exons –noadj.” Differential gene expression was calculated using the HOMER command “getDiffExpression.pl.” TPM (transcript per kilobase million) were quantified for all genes matching accession number to raw counts. Differentially expressed genes were assessed with DESeq2 (*83*) at *P*adj (adjusted *P* value) < 0.05 and fold change >2 where indicated. Gene ontology enrichment analyses were performed using Metascape (*84*).

### ATAC-seq analysis

ATAC-seq FASTQ files were trimmed to 30 bp before mapping to hg38 genome with Bowtie2 with default parameters. HOMER was used to convert aligned reads into “tag directories” for further analysis. To call peaks, we used HOMER’s findPeaks with “-style factor -size 200 -minDist 200 – L 0 – C 0 -fdr 0.9.” Next, we ran irreproducibility discovery rate (IDR) pairwise on ATAC-seq for each condition. We applied IDR analysis for replicates. We used DESeq2 using HOMER’s getDIffExpression to identify differential chromatin accessibility with fold change >2 and *P*adj <0.05. The UCSC genome browser was used to visualize ATAC-seq data.

### Gene ontology pathway enrichment analysis

Gene ontology pathway enrichment analysis was performed using R. Conversion of gene symbols to EntrezID was performed using the AnnotationDbi-package (*85*). Transformation, conversion of *P* values, and ranking of genes were conducted using the tidyverse package (*86*). For enrichment of transcripts, the clusterProfiler- and the ReactomePA-package (*87*, *88*) were used. Enriched pathways were filtered for adjusted *P* values <0.05 and subsequently organized in descending order based on the normalized enrichment score. For Graphical visualization, the ggplot2-package was used (*89*).

### Treatment with okadaic acid and withaferin A

For the treatment of neurons, maturation medium was replaced on differentiation day 24 by N2B27 medium containing 25 nM okadaic acid and 2 or 5 μM withaferin A for 3 hours. DMSO was used as the vehicle control for okadaic acid treatment and ethanol for withaferin A treatment. Subsequently, the medium was discarded and cells were washed with prechilled PBS twice. Afterward, the cells were detached using prewarmed Accutase at 37°C for 5 min. Accutase was rinsed using PBS, and cells were centrifuged for 5 min at 300*g* at RT. Supernatant was discarded, and cell pellets were snap-frozen in liquid nitrogen and stored at −80°C until further processing.

### Statistical analysis

Statistical analyses were conducted using GraphPad Prism software (version 9.5.1), except for omics studies. Unless otherwise stated, all graphs depict the mean of various neuronal differentiation experiments ± SD. Unless otherwise indicated, a two-tailed unpaired *t* test was used for comparisons between two groups. For comparisons involving three or more groups or conditions, one-way or two-way analysis of variance (ANOVA) was applied, followed by Tukey’s post hoc multiple comparisons test. A *P* value <0.05 was considered statistically significant, with * < 0.05, ** < 0.01, *** < 0.005, and **** < 0.001. If statistical significance is not depicted in the figure, or described as such in the figure legend, the results are considered statistically not significant.

## Supplementary Materials

### This PDF file includes:

Supplementary Materials and Methods Figs. S1 to S8 Tables S1 to S5 References
