# Supplementary material for: Vimentin network dysregulation mediates neurite deficits in SNCA duplication Parkinson’s patient–derived midbrain neurons
Source: Sci Adv. 2025 Jun 6;11(23):eadq2742. doi: 10.1126/sciadv.adq2742 (PMC12143386; doi:10.1126/sciadv.adq2742)
Supplement: Supplementary file 1 — Supplementary Materials and Methods Figs. S1 to S8 Tables S1 to S5 References [file sciadv.adq2742_sm.pdf]

Science Advances
AAAS

# Supplementary Materials for

## Vimentin network dysregulation mediates neurite deficits in *SNCA* duplication Parkinson's patient–derived midbrain neurons

Yanni Schneider *et al.*

Corresponding author: Wei Xiang, wei.xiang@uk-erlangen.de

*Sci. Adv.* **11**, eadq2742 (2025)
DOI: 10.1126/sciadv.adq2742

**This PDF file includes:**

Supplementary Materials and Methods
Figs. S1 to S8
Tables S1 to S5
References

## Supplementary materials and methods

### LDH cell death assay

Cell death was assessed using the CyQUANT™ LDH assay (Thermo Fisher, #C20300). In brief, 50 µl medium was collected from each well of a 12-well plate on day 24 of the neuronal midbrain differentiation and immediately put on ice for further processing. Each sample was tested in triplicates. The assay was conducted according to the manufacturers protocol, and absorbance was measured in a 96-well plate at 490 nm and 680 nm. LDH activity was calculated by subtracting the 680 nm absorbance from the 490 nm absorbance value.

### In-gel digest

Gel pieces were thoroughly washed with ABC buffer containing water, acetonitrile, and 50 mM ammonium bicarbonate, and then reduced in 10 mM dithiothreitol at 56°C for 45 min. After discarding the supernatant, 55 mM iodoacetamide (IAA) in ABC buffer was added and incubated at 25˚C for 30 min. The supernatant was discarded, followed by two washes with a 1:1 mixture of acetonitrile and 50 mM ABC buffer, each at 25˚C for 15 min. Next, the gel pieces were washed with acetonitrile for 10 min at 25 ˚C, followed by drying in a SpeedVac for 10 min.
Proteins were next digested in-gel in 25 mM ABC buffer containing 0.5 µg trypsin (Promega, V5113) overnight at 37˚C, 300 rpm. Peptides were collected by transferring the supernatant into a new 1.5 Eppendorf tube. The gel pieces were further incubated in 100 µl 25 mM ABC buffer for 15 min in an ultrasonic bath, followed by addition of 100 µl acetonitrile and continued incubation in ultrasonic bath for 15 min. These fractions (200 µl in total) were added to the first portion of eluted peptides. Next, the remaining peptides in gel pieces were eluted with 100 µl of 5% formic acid in water, incubation for 15 min in ultrasonic bath, addition of 100 µl acetonitrile, incubation for 15 min in ultrasonic bath and added to the first and second elution fraction. The combined elution fractions were dried in a SpeedVac (5-10 hours) and stored at -80 ˚C. Before analysis, peptides were dissolved in 20 µl of 1% formic acid in water, vortexed, spun down, and incubated in ultrasonic bath for 10 min. Before being transferred to MassSpec vials, dissolved peptides were filtered on 0.22 µm membrane (Merck Millipore, UFC30GV00) at 13,000 rpm, for 10 min at room temperature.

### Animals and preparation of mouse brain tissue samples for WB analysis

Breeding and housing of animals as well as brain removal for WB analyses were approved by the local governmental administrations for animal health (TS03-20, Division for Animal Welfare, Friedrich-Alexander-University Erlangen-Nürnberg, and AZ. 55.2,2-2532-2-1489 "Regierung von Unterfranken, Würzburg).
The whole brain tissue samples of mice were first homogenized in homogenization buffer (50 mM Tris/HCl, 150 mM NaCl, pH 8) using a Braun Potter S Homogenizer (Sartorius AG). The homogenate was then mixed with 4 × RIPA buffer, at a ratio of 1:3 (homogenate to 4 ×RIPA buffer (200 mM Tris/HCl buffer pH7.4, 600 mM NaCl, 4% Nonidet p-40, 0.4% SDS)) and incubated on ice for 30 min. The lysate was next centrifuged at 10,000 g for 20min at 4°C. The protein concentration in the resulting supernatant was determined using BCA assay. For SDS-PAGE 20 µg total protein were loaded.

## Supplementary results: figures and tables

## Figure S1

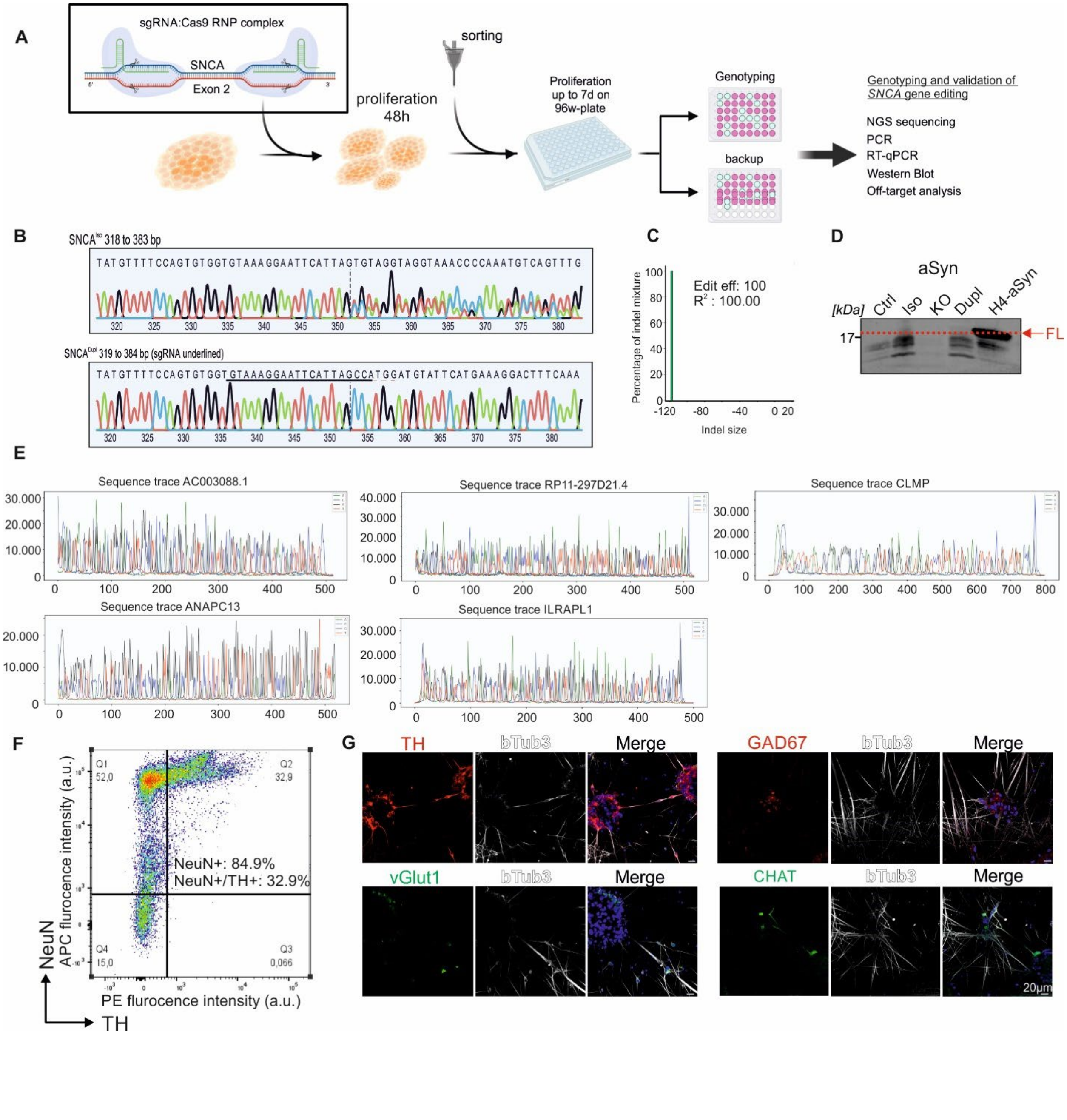


## Evaluation of CRISPR/Cas9 *SNCA* editing and analysis of neuronal differentiation efficiency.

**A)** Workflow illustrating the generation of *SNCA*$^{Iso}$ hiPSC line through CRISPR/Cas9 editing of the *SNCA* gene in the *SNCA*$^{Dupl}$ hiPSC line using the approach as described previously (24). hiPSCs were transfected with Cas9 Ribonucleoproteins (RNP) containing sgRNAs flanking *SNCA* exon 2 (Table S3) and subsequently sorted as single cells. Single hiPSC clones were then duplicated for genotyping and expanded for further analyses to assess *SNCA*

editing efficiency, including amplicon next generation sequencing (NGS), PCR amplification of the edited DNA fragment, RT-qPCR, WB, and off-target analysis. **B)** Chromatograms displaying NGS sequencing results of hiPSC clones. Lower panel: unmodified sequence of *SNCA* in the *SNCA*$^{\text{Dupl}}$ line with the sgRNA sequence highlighted (underlined). Upper panel: The edited clone demonstrates altered sequence downstream of the sgRNA sequence (starting from dashed line). Created in BioRender. Xiang, W. (2025) https://BioRender.com/059cuvk. **C)** Analysis of amplicon sequencing data from the modified *SNCA* DNA fragment extracted from agarose gel (as shown in Figure 1B) using the Inference of CRISPR Edits (ICD) tool demonstrates a 118 bp deletion with 100 percentage. Y-axis: percentage of modification, X-axis: size of indel (insert-deletion). **D)** WB of aSyn levels in hiPSCs indicating the absence of full-length (FL) aSyn at the hiPSC stage. A H4 neuroglioma cell line permanently overexpressing aSyn (18) and a *SNCA* knock-out hiPSC line (24) served as positive and negative controls of aSyn, respectively. Full-length aSyn is absent in control, *SNCA*$^{\text{Dupl}}$, and *SNCA*$^{\text{Iso}}$ hiPSC lines. **E)** Off-target analysis of the sgRNAs used for CRISPR/Cas9-mediated *SNCA* editing. Five loci (*AC003088.1, RP11-297D21.4, ANAPC13, ILRAPL1, and CLMP*) with the highest homology to the sgRNA sequences were identified using crispor.tefor.net. PCR amplification followed by DNA sequencing of these loci (24) in the *SNCA*$^{\text{Iso}}$ hiPSC line revealed no modifications, suggesting specific targeting effects of the sgRNAs used in this study. **F)** Example scatterplot from flow cytometric analysis of neuronal marker NeuN and dopaminergic marker TH in midbrain neurons differentiated from the control hiPSC line for 24 days. **G)** Representative immunofluorescence staining of neuronal subtypes in midbrain neurons differentiated from the control hiPSC line for 24 days. The upper left panel shows staining of dopaminergic neuron marker TH and neuronal marker bTubIII, indicating a larger proportion of TH-positive neurons. Staining for GAD67 (upper right panel, marker for GABAergic neurons), vGlut1 (lower left panel, marker for glutamatergic neurons) and CHAT (lower right panel, marker for cholinergic neurons) display a small proportion of these neurons in the midbrain neuron culture.

# Figure S2

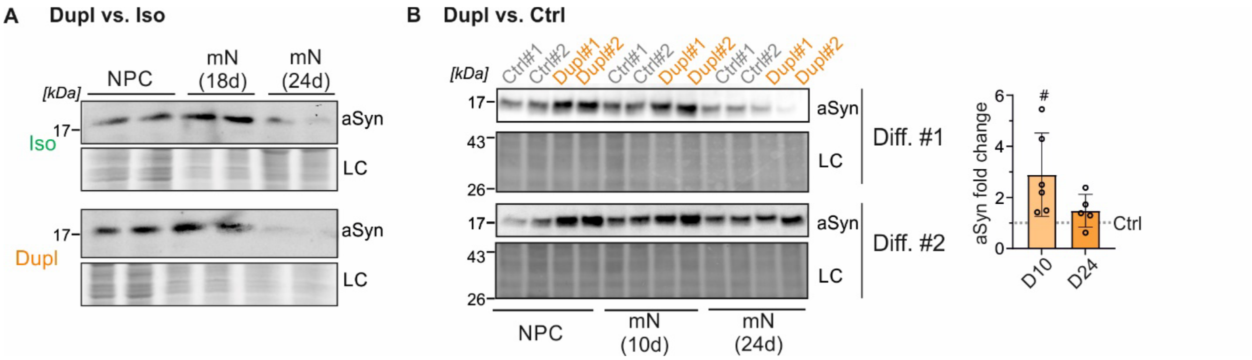


## Analysis of aSyn levels during midbrain neuronal differentiation.

WB analysis of dynamic changes in aSyn levels upon induction of differentiation, comparing *SNCA*$^{\text{Dupl}}$ with *SNCA*$^{\text{Iso}}$ (**A**) and *SNCA*$^{\text{Dupl}}$ with control cells (**B**). At the NPC stage and earlier differentiation stages (10-18 days), *SNCA*$^{\text{Dupl}}$ cells exhibit higher aSyn levels than *SNCA*$^{\text{Iso}}$ and controls, whereas this effect is reduced at an advanced differentiation stage (24 days). mN: midbrain neurons. A): WB images from N= 2 differentiation rounds. LC: Coomassie staining; B): WB images were partially presented in Seebauer et al. (18), and the data were reanalyzed and integrated into current quantification. Representative WB images from N=2 differentiation rounds. LC: Ponceau staining. For quantification in B), data from the hiPSC lines Ctrl#1, Ctrl#2, *SNCA*$^{\text{Dupl}}$#1, and *SNCA*$^{\text{Dupl}}$#2 (Table S1) with N=3 differentiation rounds for each line, were used. Unpaired t-test was performed to compare *SNCA*$^{\text{Dupl}}$ neurons differentiated for 10 days or 24 days with their corresponding control counterparts (dashed line), with significance indicated by # $p < 0.05$.

**Figure S3**

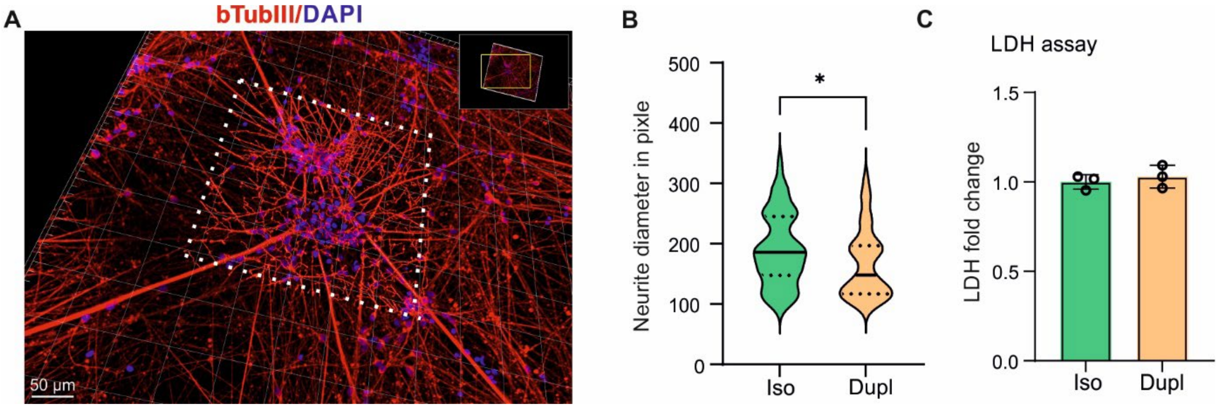


## Analysis of neurite morphology and cell death.

**A)** 3D reconstruction and neurite tracing example with IMARIS software. Neurons were immunostained for bTubIII (red). The area selected for neurite analysis within IMARIS is highlighted in white. **B)** Neurite diameter additionally analyzed using the "Simple Neurite tracer (SNT)" of Neuroanatomy plugin with the ImageJ software (FIJI distribution, v1.54f (77, 90, 91). Midbrain neurons were immunostained with an anti-bTubIII antibody and bTubIII+ primary neurites were traced and their diameter measured using SNT. Neurite diameter was analyzed on 59 and 44 bTubIII+ neurites from *SNCA*$^{\text{Iso}}$ and *SNCA*$^{\text{Dupl}}$ midbrain neurons, respectively. **C)** LDH cell death assay. Supernatant of *SNCA*$^{\text{Iso}}$ and *SNCA*$^{\text{Dupl}}$ midbrain neurons differentiated for 24 days were analyzed, showing no significant difference in cell death. N=3 of differentiation rounds.

**Figure S4**

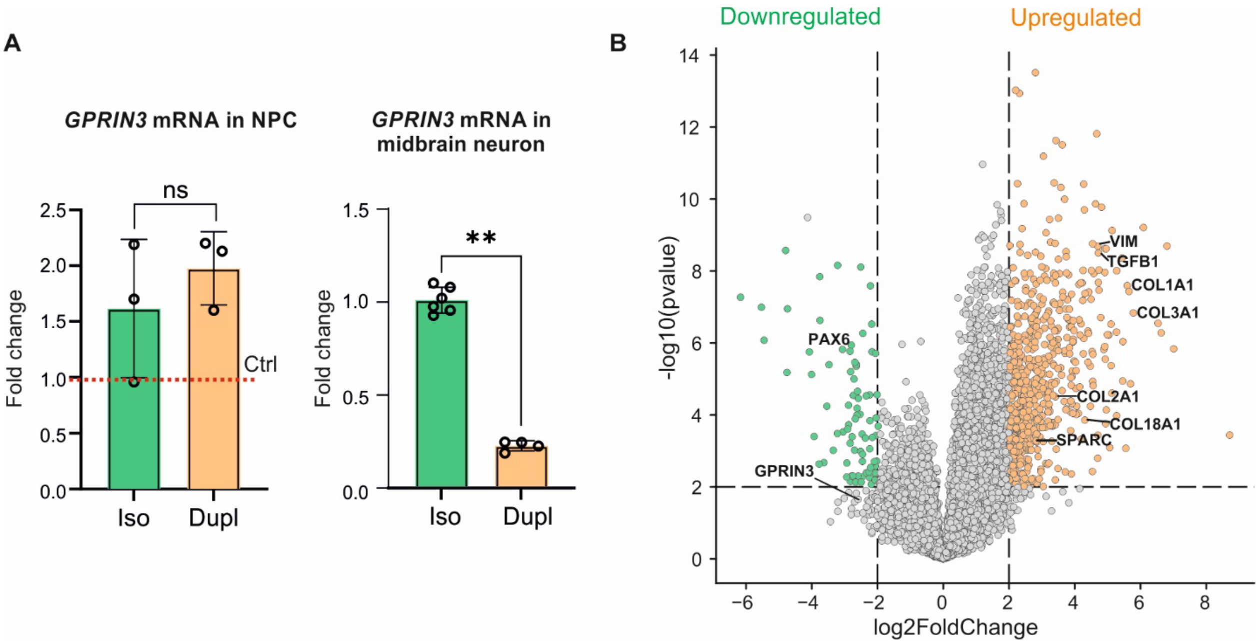


## Analysis of *GPRIN3* mRNA and overview of whole proteomic changes in *SNCA*$^{Dupl}$ midbrain neurons.

**A)** *GPRIN3* mRNA levels in NPCs and midbrain neurons. At the NPC stage, no significant difference in *GPRIN3* mRNA levels between *SNCA*$^{Dupl}$ and *SNCA*$^{Iso}$ cells is detected. Fold changes against levels in healthy donor-derived NPCs (red dashed line) are displayed. At the stage of differentiated neurons, *GPRIN3* expression is significantly reduced in *SNCA*$^{Dupl}$ neurons. mRNA levels were determined by RT-qPCR for NPCs (N=3 of differentiation rounds) and by RNA-seq analysis for midbrain neurons, respectively. For *SNCA*$^{Iso}$ neurons: N=3 differentiation rounds; for *SNCA*$^{Dupl}$ neurons: N=2 differentiation rounds, with experimental duplicates/round. **B)** Volcano plot of differentially expressed proteins in midbrain neurons identified by MassSpec proteomic analysis. Proteins involved in ECM organization (COLs), cellular differentiation and outgrowth (*PAX6*, *TGFB1, GPRIN3*), and intermediate filaments (*VIM*) are highlighted. X-axis: binary logarithm of fold change of protein (log2FoldChange); Y-axis: negative decadic logarithm of *p*-value (-log10(pvalue)). For *SNCA*$^{Iso}$ neurons: N=3 differentiation rounds, for *SNCA*$^{Dupl}$ neurons: N=2 differentiation rounds, with experimental duplicates/round.

**Figure S5**

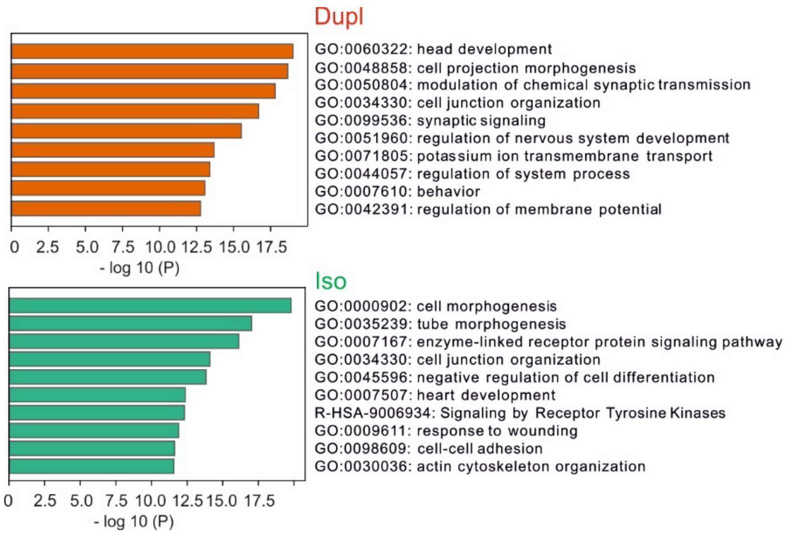


## Gene ontology pathway analysis using genes with altered chromatin accessibility.

Genes exhibiting altered chromatin accessibility between *SNCA*$^{Dupl}$ and *SNCA*$^{Iso}$ midbrain neurons were identified through ATAC-seq and subjected to gene ontology analysis. The top 10 pathways enriched with genes displaying increased chromatin accessibility in *SNCA*$^{Dupl}$ midbrain neurons (upper panel) and *SNCA*$^{Iso}$ midbrain neurons (lower panel) are illustrated. X-axis: Adjusted – log10 *p*-value; Y-axis, Identified pathways. Data were derived from N=2 differentiation rounds, with experimental duplicates/round.

**Figure S6**

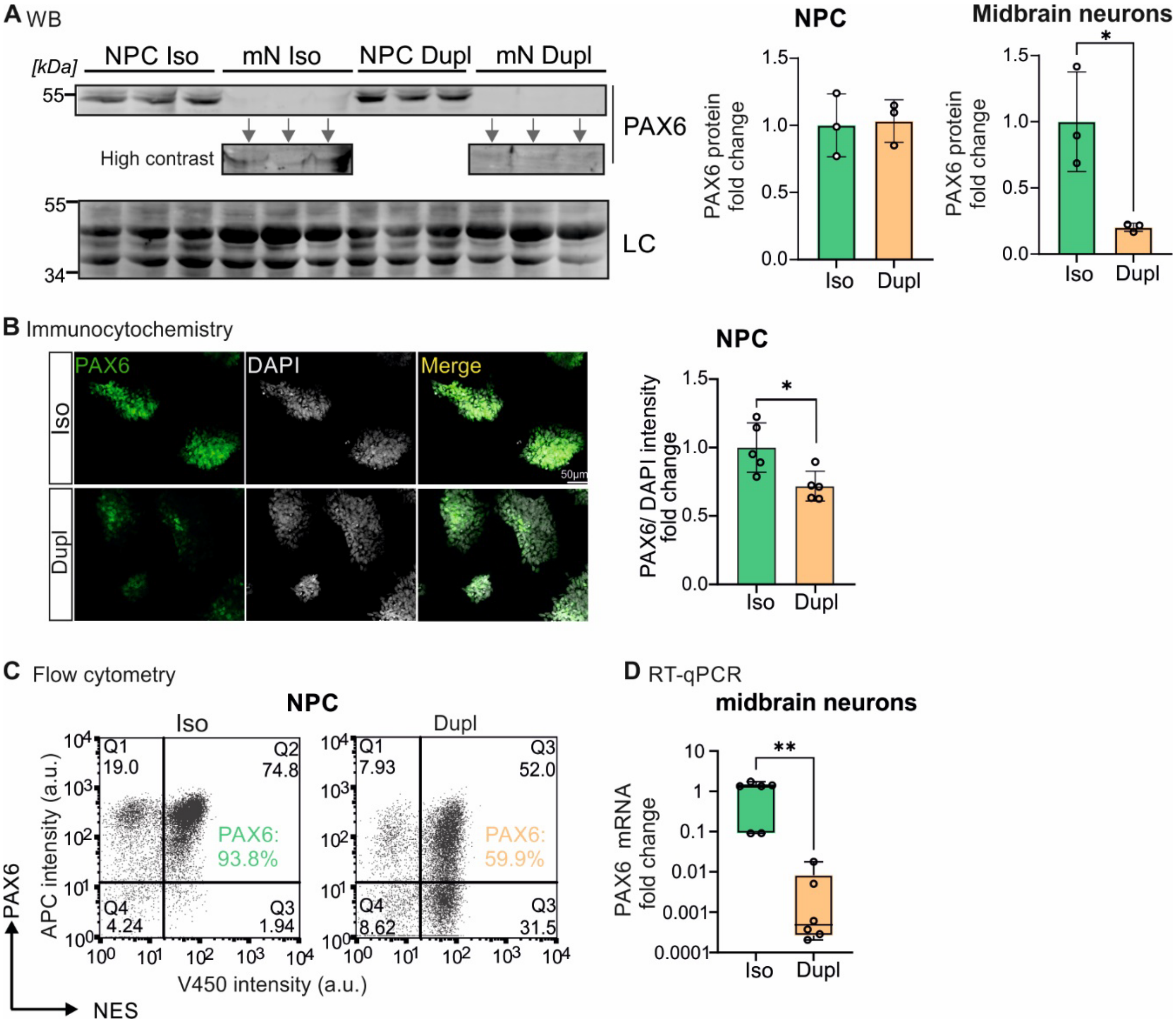


## Expression analysis of PAX6 in *SNCA*$^{Iso}$ and *SNCA*$^{Dupl}$ NPCs and midbrain neurons.

**A)** WB analysis of PAX6 levels in *SNCA*$^{Iso}$ and *SNCA*$^{Dupl}$-derived NPCs and midbrain neurons. Due to its low expression levels in midbrain neurons, PAX6 is additionally displayed with high contrast. LC: Revert 520 Total Protein Stain. N=3 independent experiments. Middle: quantification of WB, showing comparable PAX6 expression at the NPC stage. Right: quantification of WB, revealing significant downregulation of the PAX6 protein in *SNCA*$^{Dupl}$ midbrain neurons. **B)** Representative ICC images of PAX6 fluorescence staining. For quantification, the mean PAX6 signal intensity was measured by using Zen 2013 software and normalized to DAPI. The fold change in *SNCA*$^{Dupl}$ NPCs relative to *SNCA*$^{Iso}$ NPCs is shown. PAX6 intensity is significantly reduced in *SNCA*$^{Dupl}$ NPCs. Data were obtained from the analysis of five NPC clusters across N=3 independent experiments. **C)** Example scatterplot from flow cytometric analysis of NPC markers NES and PAX6. *SNCA*$^{Dupl}$ NPCs exhibit a notably reduced proportion of PAX6+ cells (59.9%) compared to *SNCA*$^{Iso}$ NPCs (93.8%). **D)** RT-qPCR analysis of *PAX6* mRNA in *SNCA*$^{Iso}$ and *SNCA*$^{Dupl}$ midbrain neurons, showing significant downregulation of *PAX6* gene expression in *SNCA*$^{Dupl}$ neurons. N=6 differentiation rounds.

# Figure S7

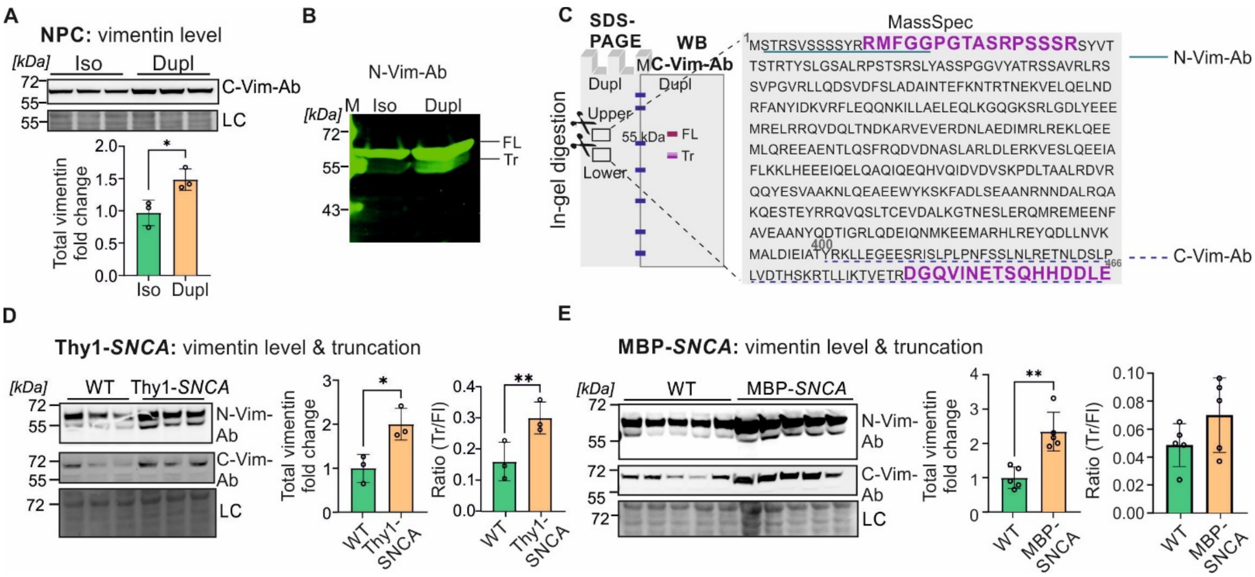


## Vimentin levels in NPCs and in animal models of synucleinopathies.

**A)** WB analysis of vimentin levels in *SNCA*$^{Iso}$ and *SNCA*$^{Dupl}$ NPCs using the C-terminal vimentin antibody. Vimentin levels were significantly elevated in *SNCA*$^{Dupl}$ NPCs compared to *SNCA*$^{Iso}$ NPCs. LC: Ponceau staining. N=3 NPC differentiation rounds. **B)** WB image of *SNCA*$^{Iso}$ and *SNCA*$^{Dupl}$ neurons analyzed by using the N-terminal vimentin antibody. Extended run time for SDS-PAGE resolves a truncated vimentin band at 55kDa in both *SNCA*$^{Iso}$ and *SNCA*$^{Dupl}$ neurons. M: protein standard marker (Blue Prestained Protein Standard, Broad Range from New England Biolabs, #P7718S). **C)** Illustration of the in-gel digestion workflow to study vimentin truncation in *SNCA*$^{Dupl}$ neurons via MassSpec analysis. Lysates from *SNCA*$^{Dupl}$ neurons were separated via SDS-PAGE in duplicates, with the protein standard (M) placed between the lanes. The right half of the gel, containing one replicate, was transferred onto a PVDF membrane and probed with the anti-C-terminal vimentin antibody to visualize the positions of full-length (FL) and truncated (Tr) vimentin. The membrane was then aligned with the left half of the gel and used as stencil to excise corresponding gel pieces carrying the respective vimentin forms. The gel pieces were next subjected to in-gel trypsin digestion followed by MassSpec analysis. The primary amino acid sequence of vimentin is shown on the right, with the missing N- and C-terminal tryptic fragments highlighted in purple (see also Table S5). Epitopes for the two antibodies used in this study targeting the N- and C-termini of vimentin are underlined, while the dashed underline indicates the approximate binding site of the C-terminal vimentin antibody. Left panel: Created in BioRender. Xiang, W. (2025) https://BioRender.com/ft1l98f. **D)** and **E)** WB analysis of vimentin levels in whole brain lysates from 6-month-old male Thy1-*SNCA* PD animals (D) and from MBP-*SNCA* MSA animals at 4 weeks old (E), compared to their wildtype (WT) non-transgenic counterparts. In both synucleinopathy animal models based on the overexpression of human aSyn, transgenic mice exhibit a significant increase in total vimentin levels compared to their wildtype littermates, as detected using vimentin antibodies targeting either the N- (N-Vim-ab) or C-terminal (C-Vim-ab) vimentin. Additionally, WB with the N-terminal vimentin antibody revealed a significant increase in vimentin truncation in Thy1-*SNCA* animals (D, right), while a trend toward an increase was observed in MBP-*SNCA* mice (E, right). WB data from staining with the N-terminal vimentin antibody were used for quantification. LC: Ponceau staining. N=3 Thy1-*SNCA* mice and their wild type littermates, as well as N=5 MBP-*SNCA* mice and their wildtype littermates.

## Figure S8

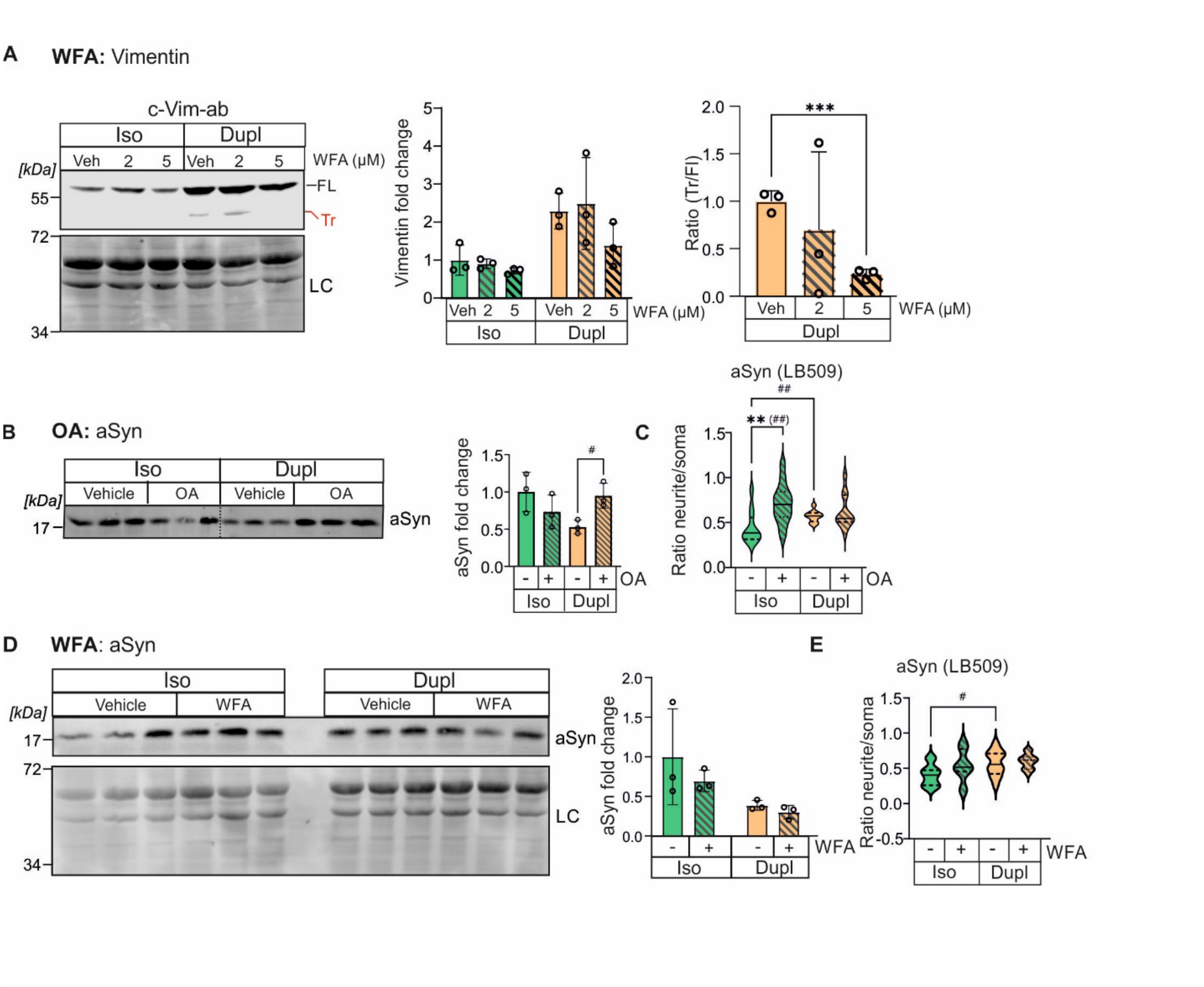


**Effect of vimentin interference on vimentin and aSyn.** **A)** WB analysis of vimentin levels in *SNCA*$^{Iso}$ and *SNCA*$^{Dupl}$ midbrain neurons after treatment with withaferin A (WFA). Total vimentin levels are not affected by withaferin A treatment (middle). *SNCA*$^{Dupl}$ neurons show significant dose-dependent reduction in the ratio of truncated (Tr)/full-length (Fl) vimentin in response to withaferin A treatment. **B)** WB analysis of aSyn levels upon treatment with okadaic acid (OA) in midbrain neurons. The treatment leads to a significant increase in aSyn levels in *SNCA*$^{Dupl}$ neurons compared to vehicle-treated *SNCA*$^{Dupl}$ neurons. **C)** ICC analysis of aSyn distribution using the LB509 antibody. Okadaic acid treatment leads to a significant redistribution of aSyn from the soma to the neurites in *SNCA*$^{Iso}$ neurons, while no changes are observed in *SNCA*$^{Dupl}$ neurons. For quantification, data from 12 neurons/group from N=3 differentiation rounds were used. **D)** WB analysis of aSyn levels upon withaferin A (2 µM) treatment in *SNCA*$^{Iso}$ and *SNCA*$^{Dupl}$ midbrain neurons. Upon treatment, aSyn levels are not significantly changed compared vehicle-treated neurons. **E)** ICC analysis of aSyn distribution using the LB509 antibody. No significant changes in aSyn distribution between the soma and neurites are observed in both *SNCA*$^{Iso}$ and *SNCA*$^{Dupl}$ neurons treated with withaferin A (2 µM) comparted to respective vehicle-treated neurons. For quantification, 12 neurons/group from N=3 differentiation rounds were analyzed. Statistical analyses in C and E, in addition to ANOVA analysis followed by Tukey's multiple comparisons for comparisons between multiple groups as detailed in the Methods section (* or **), # or ## resulted from unpaired t-tests for comparisons between the indicated two groups, with # $p < 0.05$, ## < 0.01.

## Table S1. hiPSC cell lines used in this study

| hiPSC clone code | hiPSC clone ID | Donor code | Diagnose | Sex | Age | *SNCA* mutation |
|---|---|---|---|---|---|---|
| Ctrl#1 | UKERiG3G-R1-039 | *1 | Control | M | 69 | N/A |
| Ctrl#2 | UKERi1JF-R1-011 | *2 | Control | F | 42 | N/A |
| Ctrl#3 | UKERi1JF-R1-018 | | | | | |
| *SNCA*$^{Dupl}$#1 | UKERi7GG-S1-004 | *3 | PD with *SNCA* duplication | F | 44 | N/A |
| *SNCA*$^{Dupl}$#2 | UKERi7GG-S1-005 | | | | | |
| *SNCA*$^{Iso}$ | UKERi7GG-S1-004-1 | N/A | N/A | N/A | N/A | CRISPR/Cas 9 corrected, from *SNCA*$^{Dupl}$#1 |

**Table S2. Demographic and clinical data of *post-mortem* tissue donors analyzed in this study**

| Diagnosis | Sex | Age (yrs) | *Post-mortem* (min) | Brain weight (g) | Braak NF score | Braak Lewy stage |
|---|---|---|---|---|---|---|
| Control#1 | M | 77 | 507 | 1319 | II | 0 |
| Control#2 | M | 77 | 435 | 1295 | I | 0 |
| Control#3 | F | 81 | 444 | 1180 | II | 0 |
| Control#4 | M | 92 | 435 | 1360 | III | 0 |
| Control#5 | F | 80 | 507 | 1220 | III | 0 |
| PD#1 | M | 77 | 429 | 1285 | III | 6 |
| PD#2 | F | 82 | 550 | 1162 | II | 6 |
| PD#3 | M | 77 | 381 | 1355 | II | 6 |
| PD#4 | F | 79 | 207 | 1027 | II | 5 |
| PD#4 | M | 92 | 606 | 1400 | IV | 5 |

**Table S3. List of primers and oligonucleotides used in this study**

| Gene | Primer pair |
|---|---|
| *GPRIN3* | F: ACACACTGGAGCTCTCTGAC<br><br>R: CTGGGCATTACTGGAGACCA |
| *SNCA*<br>(RT-qPCR*)* | F: CCATGGATGTATTCATGAAAGGACT<br><br>R: AAGTGGTCGTTGAGGGCAATG |
| 18S | F: GGAGTATGGTTGCCAAGCTGA<br><br>R: ATCTGTCAATCCTGTCCGTGT |
| *GAPDH* | F: CTGGGCTACACTGAGCACC<br><br>R: AAGTGGTCGTTGAGGGCAATG |
| *PAX6* | F: CAGGGCAATCGGTGGTAGTA<br><br>R: CTGTTTATTGATGACACGCTTGG |
| sgRNA<br>oligonucleotides | F: GTAAAGGAATTCATTAGCCA<br><br>R: AAAGAGGGTGTTCTCTATGT |
| SNCA<br>(PCR and<br>sequencing) | F: CCTTCTGCCTTTCCACCCT<br><br>R: TTGTCAGGATCCACAGGCAT |

**Table S4. List of antibodies used in this study**

| Target | Source | Company | Catalog Nr. | RRID | Application/dilution |
|---|---|---|---|---|---|
| aSyn (Syn1) | Mouse | BD Biosciences | 610786 | AB_398108 | WB/ 1:1000 |
| aSyn (LB509) | Mouse | Abcam | ab27766 | AB_727020 | ICC/ 1:50 |
| aSyn (15G7) | Rat | Enzo Lifesciences | ALX-804-258-L001 | AB_2270759 | ICC/ 1:100 |
| CHAT | Goat | Millipore | AB144P | AB_2079751 | ICC/ 1:500 |
| GAD67 | Mouse | Millipore | MAB5406 | AB_2278725 | ICC/ 1:500 |
| NES | Mouse | Millipore | MAB5326 | AB_11211837 | ICC/ 1:400 |
| NES, VF 450 conjugated | Mouse | BD Biosciences | 561551 | AB_10717122 | FC/ 1:75 |
| SOX2 | Rabbit | Cell Signaling | 3579 | AB_2195767 | ICC/ 1:400 |
| PAX6, APC-conjugated | Human | Miltenyi | 130-123-328 | AB_2819477 | FC/ 1:75 |
| PAX6 | Rabbit | Biolegend | 901302 | AB_2565003 | WB/ 1:500 |
| bTubIII | Mouse | BioLegend | 801201 | AB_2313773 | WB/ 1:1000<br>ICC/ 1:500 |
| bTubIII | Rabbit | BioLegend | 802001 | AB_2564645 | ICC/ 1:500 |
| TH | Rabbit | Millipore | AB152 | AB_390204 | ICC/ 1:300 |
| TH, PE-conjugated | Mouse | Miltenyi Biotech | 130-120-351 | AB_2857529 | FC/ 1:75 |
| NEUN, Alexa Fluor 647 conjugated | Rabbit | Abcam | Ab190565 | AB_2732785 | FC/ 1:75 |
| Vimentin (N-Vim) | Guinea pig | Progen | GP59 | N/A | WB/ 1:1000 |
| Vimentin (C-VIM | Rabbit | Abcam | Ab45939 | AB_2257290 | WB/ 1:1000<br>ICC/ 1:500 |
| vGlut1 | Guinea Pig | Synaptic Systems | 135 318 | N/A | ICC/ 1:500 |
| Mouse Alexa-647 | Donkey | Thermo Fisher Scientific | A-31571 | AB_162542 | WB/ 1:1000<br>ICC/ 1:500 |
| Mouse Alexa-568 | Donkey | Thermo Fisher Scientific | A10037 | AB_2534013 | WB/ 1:1000<br>ICC/ 1:500 |
| Mouse Alexa-488 | Donkey | Molecular Probes | A-21202 | AB_141607 | WB/ 1:1000<br>ICC/ 1:500 |
| Rabbit Alexa-647 | Donkey | Jackson ImmunoResearch | 711-605-152 | AB_2492288 | WB/ 1:1000<br>ICC/ 1:500 |
| Rabbit Alexa-568 | Donkey | Thermo Fisher Scientific | A10042 | AB_2534017 | WB/ 1:1000<br>ICC/ 1:500 |
| Rabbit Alexa-488 | Donkey | Molecular Probes | A-21206 | AB_2535792 | WB/ 1:1000<br>ICC/ 1:500 |
| Rat Alexa-568 | Goat | Thermo Fisher Scientific | A-11077 | AB_2534121 | WB/ 1:1000<br>ICC/ 1:500 |
| Guinea pig Alexa-488 | Donkey | Jackson ImmunoResearch | 706-545-148 | AB_2340472 | WB/ 1:1000 |

| | | | | | |
|---|---|---|---|---|---|
| Rabbit IRDye 800CW | Donkey | Licor | 926-32213 | AB_621848 | WB/ 1:10000 |
| Mouse IRDye 680RD | Donkey | Licor | 926-68072 | AB_10953628 | WB/ 1:10000 |

**Table S5. In-gel tryptic digest analysis of vimentin truncation**

| Replicate | 1 | | 2 | |
|---|---|---|---|---|
| | upper gel piece (FL) | lower gel piece (< 55 kDa) | upper gel piece (FL) | lower gel piece (< 55 kDa |
| **N-terminal peptide** | | | | |
| RMFGGPGTASRPSSSR | 521436 | | 800828 | |
| **C-terminal peptides** | | | | |
| LQDEIQNMK | 6.86E+07 | 1.60E+06 | 1.04E+08 | 7.78E+06 |
| MALDIEIATYR | 4.48E+06 | 1.46E+07 | 5.14E+06 | 1.19E+06 |
| ETNLDSLPLVDTHSK | 2.38E+08 | 7.60E+06 | 6.52E+08 | 9.76E+07 |
| ETNLDSLPLVDTHSKR | 6.72E+06 | | 1.17E+07 | 4.14E+06 |
| TVETRDGQVINETSQHHDDLE | 6.28E+06 | | 1.38E+07 | 2.02E+06 |
| DGQVINETSQHHDDLE | 1.13E+06 | | 2.46E+06 | |

The table contains identified and quantified peptides from the upper gel piece containing the full-length (FL) vimentin and the lower gel piece containing truncated vimentin forms < 55kDa, as shown in Figure S7C. In-gel digestion was performed in duplicates. The values represent MaxLFQ normalized intensities (92).

## REFERENCES AND NOTES


1. M. G. Spillantini, M. L. Schmidt, V. M. Lee, J. Q. Trojanowski, R. Jakes, M. Goedert, Alpha-synuclein in Lewy bodies. *Nature* **388**, 839–840 (1997).

2. M. Baba, S. Nakajo, P. H. Tu, T. Tomita, K. Nakaya, V. M. Lee, J. Q. Trojanowski, T. Iwatsubo, Aggregation of alpha-synuclein in Lewy bodies of sporadic Parkinson's disease and dementia with Lewy bodies. *Am. J. Pathol.* **152**, 879–884 (1998).

3. C. Klein, A. Westenberger, Genetics of Parkinson’s disease. *Cold Spring Harb. Perspect. Med.* **2**, a008888 (2012).

4. L. Maroteaux, J. T. Campanelli, R. H. Scheller, Synuclein: A neuron-specific protein localized to the nucleus and presynaptic nerve terminal. *J. Neurosci.* **8**, 2804–2815 (1988).

5. J. Burré, M. Sharma, T. Tsetsenis, V. Buchman, M. R. Etherton, T. C. Südhof, Alpha-synuclein promotes SNARE-complex assembly in vivo and in vitro. *Science* **329**, 1663–1667 (2010).

6. L. Wang, U. Das, D. A. Scott, Y. Tang, P. J. McLean, S. Roy, α-Synuclein multimers cluster synaptic vesicles and attenuate recycling. *Curr. Biol.* **24**, 2319–2326 (2014).

7. J. Sun, L. Wang, H. Bao, S. Premi, U. Das, E. R. Chapman, S. Roy, Functional cooperation of α-synuclein and VAMP2 in synaptic vesicle recycling. *Proc. Natl. Acad. Sci. U.S.A.* **116** 11113–11115 (2019).

8. S. Liu, I. Ninan, I. Antonova, F. Battaglia, F. Trinchese, A. Narasanna, N. Kolodilov, W. Dauer, R. D. Hawkins, O. Arancio, α-Synuclein produces a long-lasting increase in neurotransmitter release. *EMBO J.* **23**, 4506–4516 (2004).

9. I. Gureviciene, K. Gurevicius, H. Tanila, Role of alpha-synuclein in synaptic glutamate release. *Neurobiol. Dis.* **28**, 83–89 (2007).

10. C. Y. Chung, J. B. Koprich, H. Siddiqi, O. Isacson, Dynamic changes in presynaptic and axonal transport proteins combined with striatal neuroinflammation precede dopaminergic neuronal loss in a rat model of AAV alpha-synucleinopathy. *J. Neurosci.* **29**, 3365–3373 (2009).

11. Y. Chu, G. A. Morfini, L. B. Langhamer, Y. He, S. T. Brady, J. H. Kordower, Alterations in axonal transport motor proteins in sporadic and experimental Parkinson's disease. *Brain* **135**, 2058–2073 (2012).

12. J. Grosch, J. Winkler, Z. Kohl, Early degeneration of both dopaminergic and serotonergic axons - A common mechanism in Parkinson's disease. *Front. Cell. Neurosci.* **10**, 293 (2016).

13. F. Zambon, M. Cherubini, H. J. R. Fernandes, C. Lang, B. J. Ryan, V. Volpato, N. Bengoa-Vergniory, S. Vingill, M. Attar, H. D. E. Booth, W. Haenseler, J. Vowles, R. Bowden, C. Webber, S. A. Cowley, R. Wade-Martins, Cellular alpha-synuclein pathology is associated with bioenergetic dysfunction in Parkinson's iPSC-derived dopamine neurons. *Hum. Mol. Genet.* **28**, 2001–2013 (2019).

14. I. Stojkovska, W. Y. Wani, F. Zunke, N. R. Belur, E. A. Pavlenko, N. Mwenda, K. Sharma, L. Francelle, J. R. Mazzulli, Rescue of α-synuclein aggregation in Parkinson's patient neurons by synergistic enhancement of ER proteostasis and protein trafficking. *Neuron*, 436–451.e11 2021.

15. A. Drobny, F. A. Boros, D. Balta, S. Prieto Huarcaya, D. Caylioglu, N. Qazi, J. Vandrey, Y. Schneider, J. P. Dobert, C. Pitcairn, J. R. Mazzulli, F. Zunke, Reciprocal effects of alpha-synuclein aggregation and lysosomal homeostasis in synucleinopathy models. *Transl. Neurodegener.* **12**, 31 (2023).

16. L. M. A. Oliveira, L. J. Falomir-Lockhart, M. G. Botelho, K.-H. Lin, P. Wales, J. C. Koch, E. Gerhardt, H. Taschenberger, T. F. Outeiro, P. Lingor, B. Schüle, D. J. Arndt-Jovin, T. M. Jovin, Elevated α-synuclein caused by SNCA gene triplication impairs neuronal differentiation and maturation in Parkinson's patient-derived induced pluripotent stem cells. *Cell Death Dis.* **6**, e1994 (2015).

17. J. C. Koch, F. Bitow, J. Haack, Z. d'Hedouville, J.-N. Zhang, L. Tönges, U. Michel, L. M. Oliveira, T. M. Jovin, J. Liman, L. Tatenhorst, M. Bähr, P. Lingor, Alpha-synuclein affects neurite morphology, autophagy, vesicle transport and axonal degeneration in CNS neurons. *Cell Death Dis.* **6**, e1811 (2015).

18. L. Seebauer, Y. Schneider, A. Drobny, S. Plötz, T. Koudelka, A. Tholey, I. Prots, B. Winner, F. Zunke, J. Winkler, W. Xiang, Interaction of alpha synuclein and microtubule organization is linked to impaired neuritic integrity in Parkinson's patient-derived neuronal cells. *Int. J. Mol. Sci.* **23**, 1812 (2022).

19. I. Prots, J. Grosch, R.-M. Brazdis, K. Simmnacher, V. Veber, S. Havlicek, C. Hannappel, F. Krach, M. Krumbiegel, O. Schütz, A. Reis, W. Wrasidlo, D. R. Galasko, T. W. Groemer, E. Masliah, U. Schlötzer-Schrehardt, W. Xiang, J. Winkler, B. Winner, α-Synuclein oligomers induce early axonal dysfunction in human iPSC-based models of synucleinopathies. *Proc. Natl. Acad. Sci. U.S.A.* **115**, 7813–7818 (2018).

20. K. Pieger, V. Schmitt, C. Gauer, N. Gießl, I. Prots, B. Winner, J. Winkler, J. H. Brandstätter, W. Xiang, Translocation of distinct alpha synuclein species from the nucleus to neuronal processes during neuronal differentiation. *Biomolecules* **12**, 1108 (2022).

21. S. Olgiati, A. Thomas, M. Quadri, G. J. Breedveld, J. Graafland, H. Eussen, H. Douben, A. de Klein, M. Onofrj, V. Bonifati, Early-onset parkinsonism caused by alpha-synuclein gene triplication: Clinical and genetic findings in a novel family. *Parkinsonism Relat. Disord.* **21**, 981–986 (2015).

22. A. Iannielli, M. Luoni, S. G. Giannelli, R. Ferese, G. Ordazzo, M. Fossati, A. Raimondi, F. Opazo, O. Corti, J. H. M. Prehn, S. Gambardella, R. Melki, V. Broccoli, Modeling native and seeded synuclein aggregation and related cellular dysfunctions in dopaminergic neurons derived by a new set of isogenic iPSC lines with SNCA multiplications. *Cell Death Dis.* **13**, 881 (2022).

23. F. Zafar, V. Nallur Srinivasaraghavan, M. Yang Chen, C. A. M. Torres, B. Schüle, Isogenic human SNCA gene dosage induced pluripotent stem cells to model Parkinson's disease. *Stem Cell Res.* **60**, 102733 (2022).

24. Y. Schneider, S. Turan, A. Koller, M. Krumbiegel, M. Farrell, S. Plötz, J. Winkler, W. Xiang, Generation of a homozygous and a heterozygous SNCA gene knockout human-induced pluripotent stem cell line by CRISPR/Cas9 mediated allele-specific tuning of SNCA expression. *Stem Cell Res.* **65**, 102952 (2022).

25. P. Reinhardt, M. Glatza, K. Hemmer, Y. Tsytsyura, C. S. Thiel, S. Höing, S. Moritz, J. A. Parga, L. Wagner, J. M. Bruder, G. Wu, B. Schmid, A. Röpke, J. Klingauf, J. C. Schwamborn, T. Gasser, H. R. Schöler, J. Sterneckert, Derivation and expansion using only small molecules of human neural progenitors for neurodegenerative disease modeling. *PLOS ONE* **8**, e59252 (2013).

26. L. Stefanis, α-Synuclein in Parkinson's disease. *Cold Spring Harb. Perspect. Med.* **2**, a009399 (2012).

27. F. Cheng, W. Zheng, C. Liu, P. A. Barbuti, L. Yu-Taeger, N. Casadei, J. Huebener-Schmid, J. Admard, K. Boldt, K. Junger, M. Ueffing, H. Houlden, M. Sharma, R. Kruger, K. Grundmann-Hauser, T. Ott, O. Riess, Intronic enhancers of the human SNCA gene predominantly regulate its expression in brain in vivo. *Sci. Adv.* **8**, eabq6324 (2022).

28. I. Stojkovska, J. R. Mazzulli, Detection of pathological alpha-synuclein aggregates in human iPSC-derived neurons and tissue. *STAR Protoc.* **2**, 100372 (2021).

29. Z. Ostrowska-Podhorodecka, I. Ding, M. Norouzi, C. A. McCulloch, Impact of vimentin on regulation of cell signaling and matrix remodeling. *Front. Cell Dev. Biol.* **10**, 869069 (2022).

30. D. Karadurmus, D. Rial, J. F. De Backer, D. Communi, A. de Kerchove d'Exaerde, S. N. Schiffmann, GPRIN3 controls neuronal excitability, morphology, and striatal-dependent behaviors in the indirect pathway of the striatum. *J. Neurosci.* **39**, 7513–7528 (2019).

31. K. R. Pennypacker, AP-1 transcription factor complexes in CNS disorders and development. *J. Fla. Med. Assoc.* **82**, 551–554 (1995).

32. L. F. Hiew, C. H. Poon, H. Z. You, L. W. Lim, TGF-β/Smad signalling in neurogenesis: Implications for neuropsychiatric diseases. *Cells* **10**, 1382 (2021).

33. A. Domanskyi, H. Alter, M. A. Vogt, P. Gass, I. A. Vinnikov, Transcription factors Foxa1 and Foxa2 are required for adult dopamine neurons maintenance. *Front. Cell. Neurosci.* **8**, 275 (2014).

34. D. Sugiaman-Trapman, M. Vitezic, E. M. Jouhilahti, A. Mathelier, G. Lauter, S. Misra, C. O. Daub, J. Kere, P. Swoboda, Characterization of the human RFX transcription factor family by regulatory and target gene analysis. *BMC Genomics* **19**, 181 (2018).

35. E. Leyva-Díaz, CUT homeobox genes: Transcriptional regulation of neuronal specification and beyond. *Front. Cell. Neurosci.* **17**, 1233830 (2023).

36. F. Sarayloo, P. A. Dion, G. A. Rouleau, MEIS1 and restless legs syndrome: A comprehensive review. *Front. Neurol.* **10**, 935 (2019).

37. J. Wen, Q. Hu, M. Li, S. Wang, L. Zhang, Y. Chen, L. Li, Pax6 directly modulate Sox2 expression in the neural progenitor cells. *Neuroreport* **19**, 413–417 (2008).

38. S. Thakurela, N. Tiwari, S. Schick, A. Garding, R. Ivanek, B. Berninger, V. K. Tiwari, Mapping gene regulatory circuitry of Pax6 during neurogenesis. *Cell Discov.* **2**, 15045 (2016).

39. J. Hsieh, Orchestrating transcriptional control of adult neurogenesis. *Genes Dev.* **26**, 1010–1021 (2012).

40. J. T. Yabe, W. K. Chan, F. S. Wang, A. Pimenta, D. D. Ortiz, T. B. Shea, Regulation of the transition from vimentin to neurofilaments during neuronal differentiation. *Cell Motil. Cytoskeleton* **56**, 193–205 (2003).

41. E. Rockenstein, M. Mallory, M. Hashimoto, D. Song, C. W. Shults, I. Lang, E. Masliah, Differential neuropathological alterations in transgenic mice expressing α-synuclein from the platelet-derived growth factor and Thy-1 promoters. *J. Neurosci. Res.* **68**, 568–578 (2002).

42. C. W. Shults, E. Rockenstein, L. Crews, A. Adame, M. Mante, G. Larrea, M. Hashimoto, D. Song, T. Iwatsubo, K. Tsuboi, E. Masliah, Neurological and neurodegenerative alterations in a transgenic mouse model expressing human α-synuclein under oligodendrocyte promoter: Implications for multiple system atrophy. *J. Neurosci.* **25**, 10689–10699 (2005).

43. W. C. Lee, J. S. Yu, S. D. Yang, Y. K. Lai, Reversible hyperphosphorylation and reorganization of vimentin intermediate filaments by okadaic acid in 9L rat brain tumor cells. *J. Cell. Biochem.* **49**, 378–393 (1992).

44. J. T. Thaiparambil, L. Bender, T. Ganesh, E. Kline, P. Patel, Y. Liu, M. Tighiouart, P. M. Vertino, R. D. Harvey, A. Garcia, A. I. Marcus, Withaferin A inhibits breast cancer invasion and metastasis at sub-cytotoxic doses by inducing vimentin disassembly and serine 56 phosphorylation. *Int. J. Cancer* **129**, 2744–2755 (2011).

45. P. Bargagna-Mohan, A. Hamza, Y. E. Kim, Y. K. A. Ho, N. Mor-Vaknin, N. Wendschlag, J. Liu, R. M. Evans, D. M. Markovitz, C. G. Zhan, K. B. Kim, R. Mohan, The tumor inhibitor and antiangiogenic agent withaferin A targets the intermediate filament protein vimentin. *Chem. Biol.* **14**, 623–634 (2007).

46. R. M. Brazdis, J. E. Alecu, D. Marsch, A. Dahms, K. Simmnacher, S. Lorentz, A. Brendler, Y. Schneider, F. Marxreiter, L. Roybon, B. Winner, W. Xiang, I. Prots, Demonstration of brain region-specific neuronal vulnerability in human iPSC-based model of familial Parkinson's disease. *Hum. Mol. Genet.* **29**, 1180–1191 (2020).

47. H. Fukusumi, K. Togo, M. Sumida, M. Nakamori, S. Obika, K. Baba, T. Shofuda, D. Ito, H. Okano, H. Mochizuki, Y. Kanemura, Alpha-synuclein dynamics in induced pluripotent stem cell-derived dopaminergic neurons from a Parkinson's disease patient (*PARK4*) with *SNCA* triplication. *FEBS Open Bio.* **11**, 354–366 (2021).

48. L. A. Volpicelli-Daley, Effects of α-synuclein on axonal transport. *Neurobiol. Dis.* **105**, 321–327 (2017).

49. M. Farrer, J. Kachergus, L. Forno, S. Lincoln, D. S. Wang, M. Hulihan, D. Maraganore, K. Gwinn-Hardy, Z. Wszolek, D. Dickson, J. W. Langston, Comparison of kindreds with parkinsonism and alpha-synuclein genomic multiplications. *Ann. Neurol.* **55**, 174–179 (2004).

50. S. Ochi, S. Manabe, T. Kikkawa, N. Osumi, Thirty years' history since the discovery of Pax6: From central nervous system development to neurodevelopmental disorders. *Int. J. Mol. Sci.* **23**, 6115 (2022).

51. T. Vitalis, O. Cases, D. Engelkamp, C. Verney, D. J. Price, Defect of tyrosine hydroxylase-immunoreactive neurons in the brains of mice lacking the transcription factor Pax6. *J. Neurosci.* **20**, 6501–6516 (2000).

52. M. G. Thomas, C. Welch, L. Stone, P. Allan, R. A. Barker, R. B. White, PAX6 expression may be protective against dopaminergic cell loss in Parkinson's disease. *CNS Neurol. Disord. Drug Targets* **15**, 73–79 (2016).

53. R. Pinho, I. Paiva, K. G. Jercic, L. Fonseca-Ornelas, E. Gerhardt, C. Fahlbusch, P. Garcia-Esparcia, C. Kerimoglu, M. A. S. Pavlou, A. Villar-Pique, E. Szego, T. Lopes da Fonseca, F. Odoardi, S. Soeroes, A. C. Rego, W. Fischle, J. C. Schwamborn, T. Meyer, S. Kugler, I. Ferrer, J. Attems, A. Fischer, S. Becker, M. Zweckstetter, F. Borovecki, T. F. Outeiro, Nuclear localization and phosphorylation modulate pathological effects of alpha-synuclein. *Hum. Mol. Genet.* **28**, 31–50 (2019).

54. A. Surguchov, α-Synuclein and mechanisms of epigenetic regulation. *Brain Sci.* **13**, 150 (2023).

55. E. Kontopoulos, J. D. Parvin, M. B. Feany, α-Synuclein acts in the nucleus to inhibit histone acetylation and promote neurotoxicity. *Hum. Mol. Genet.* **15**, 3012–3023 (2006).

56. A. J. Schaser, V. R. Osterberg, S. E. Dent, T. L. Stackhouse, C. M. Wakeham, S. W. Boutros, L. J. Weston, N. Owen, T. A. Weissman, E. Luna, J. Raber, K. C. Luk, A. K. McCullough, R. L. Woltjer, V. K. Unni, Alpha-synuclein is a DNA binding protein that modulates DNA repair with implications for Lewy body disorders. *Sci. Rep.* **9**, 10919 (2019).

57. A. Book, I. Guella, T. Candido, A. Brice, N. Hattori, B. Jeon, M. J. Farrer, SNCA Multiplication Investigators of the GEoPD Consortium, A meta-analysis of α-synuclein multiplication in familial parkinsonism. *Front. Neurol.* **9**, 1021 (2018).

58. K. Z. Chen, S. X. Liu, Y. W. Li, T. He, J. Zhao, T. Wang, X. X. Qiu, H. F. Wu, Vimentin as a potential target for diverse nervous system diseases. *Neural Regen. Res.* **18**, 969–975 (2023).

59. R. A. Nixon, T. B. Shea, Dynamics of neuronal intermediate filaments: A developmental perspective. *Cell Motil. Cytoskeleton* **22**, 81–91 (1992).

60. X. Han, J. Xu, Z. Chen, P. Li, L. Zhao, J. Tao, Y. Shen, S. Zhu, B. Yu, J. Zhu, Q. Cao, S. Zhou, Gas5 inhibition promotes the axon regeneration in the adult mammalian nervous system. *Exp. Neurol.* **356**, 114157 (2022).

61. E. C. Levin, N. K. Acharya, J. C. Sedeyn, V. Venkataraman, M. R. D'Andrea, H. Y. Wang, R. G. Nagele, Neuronal expression of vimentin in the Alzheimer's disease brain may be part of a generalized dendritic damage-response mechanism. *Brain Res.* **1298**, 194–207 (2009).

62. N. Gajendran, S. Rajasekaran, S. N. Witt, Knocking out alpha-synuclein in melanoma cells downregulates L1CAM and decreases motility. *Sci. Rep.* **13**, 9243 (2023).

63. E. Junn, S. S. Lee, U. T. Suhr, M. M. Mouradian, Parkin accumulation in aggresomes due to proteasome impairment. *J. Biol. Chem.* **277**, 47870–47877 (2002).

64. C. S. Morrow, T. J. Porter, N. Xu, Z. P. Arndt, K. Ako-Asare, H. J. Heo, E. A. N. Thompson, D. L. Moore, Vimentin coordinates protein turnover at the aggresome during neural stem cell quiescence exit. *Cell Stem Cell* **26**, 558–568.e9 (2020).

65. C. L. Phillips, D. Fu, L. E. Herring, D. Armao, N. T. Snider, Calpain-mediated proteolysis of vimentin filaments is augmented in giant axonal neuropathy fibroblasts exposed to hypotonic stress. *Front. Cell Dev. Biol.* **10**, 1008542 (2022).

66. Y. Byun, F. Chen, R. Chang, M. Trivedi, K. J. Green, V. L. Cryns, Caspase cleavage of vimentin disrupts intermediate filaments and promotes apoptosis. *Cell Death Differ.* **8**, 443–450 (2001).

67. E. Perlson, S. Hanz, K. Ben-Yaakov, Y. Segal-Ruder, R. Seger, M. Fainzilber, Vimentin-dependent spatial translocation of an activated MAP kinase in injured nerve. *Neuron* **45**, 715–726 (2005).

68. V. Zaman, K. P. Drasites, A. Myatich, R. Shams, D. C. Shields, D. Matzelle, A. Haque, N. L. Banik, Inhibition of calpain attenuates degeneration of substantia nigra neurons in the rotenone rat model of Parkinson's disease. *Int. J. Mol. Sci.* **23**, 13849 (2022).

69. M. Diepenbroek, N. Casadei, H. Esmer, T. C. Saido, J. Takano, P. J. Kahle, R. A. Nixon, M. V. Rao, R. Melki, L. Pieri, S. Helling, K. Marcus, R. Krueger, E. Masliah, O. Riess, S. Nuber, Overexpression of the calpain-specific inhibitor calpastatin reduces human alpha-Synuclein processing, aggregation and synaptic impairment in [A30P]αSyn transgenic mice. *Hum. Mol. Genet.* **23**, 3975–3989 (2014).

70. S. J. Crocker, P. D. Smith, V. Jackson-Lewis, W. R. Lamba, S. P. Hayley, E. Grimm, S. M. Callaghan, R. S. Slack, E. Melloni, S. Przedborski, G. S. Robertson, H. Anisman, Z. Merali, D. S. Park, Inhibition of calpains prevents neuronal and behavioral deficits in an MPTP mouse model of Parkinson's disease. *J. Neurosci.* **23**, 4081–4091 (2003).

71. M. Zhao, B. Wang, C. Zhang, Z. Su, B. Guo, Y. Zhao, R. Zheng, The DJ1-Nrf2-STING axis mediates the neuroprotective effects of Withaferin A in Parkinson's disease. *Cell Death Differ.* **28**, 2517–2535 (2021).

72. M. Narayan, J. Zhang, K. Braswell, C. Gibson, A. Zitnyar, D. C. Lee, S. Varghese-Gupta, U. K. Jinwal, Withaferin A regulates LRRK2 levels by interfering with the Hsp90- Cdc37 chaperone complex. *Curr. Aging Sci.* **8**, 259–265 (2015).

73. R. Ferese, N. Modugno, R. Campopiano, M. Santilli, S. Zampatti, E. Giardina, A. Nardone, D. Postorivo, F. Fornai, G. Novelli, E. Romoli, S. Ruggieri, S. Gambardella, Four copies of SNCA responsible for autosomal dominant Parkinson's disease in two italian siblings. *Parkinsons Dis.* **2015**, 546462 (2015).

74. K. Itokawa, T. Sekine, M. Funayama, H. Tomiyama, M. Fukui, T. Yamamoto, N. Tamura, H. Matsuda, N. Hattori, N. Araki, A case of α-synuclein gene duplication presenting with head-shaking movements. *Mov. Disord.* **28**, 384–387 (2013).

75. J. Fuchs, C. Nilsson, J. Kachergus, M. Munz, E. M. Larsson, B. Schule, J. W. Langston, F. A. Middleton, O. A. Ross, M. Hulihan, T. Gasser, M. J. Farrer, Phenotypic variation in a large Swedish pedigree due to SNCA duplication and triplication. *Neurology* **68**, 916–922 (2007).

76. H. Braak, K. Del Tredici, U. Rub, R. A. de Vos, E. N. Jansen Steur, E. Braak, Staging of brain pathology related to sporadic Parkinson's disease. *Neurobiol. Aging* **24**, 197–211 (2003).

77. J. Schindelin, I. Arganda-Carreras, E. Frise, V. Kaynig, M. Longair, T. Pietzsch, S. Preibisch, C. Rueden, S. Saalfeld, B. Schmid, J. Y. Tinevez, D. J. White, V. Hartenstein, K. Eliceiri, P. Tomancak, A. Cardona, Fiji: An open-source platform for biological-image analysis. *Nat. Methods* **9**, 676–682 (2012).

78. C. S. Hughes, S. Moggridge, T. Muller, P. H. Sorensen, G. B. Morin, J. Krijgsveld, Single-pot, solid-phase-enhanced sample preparation for proteomics experiments. *Nat. Protoc.* **14**, 68–85 (2019).

79. S. Tyanova, T. Temu, P. Sinitcyn, A. Carlson, M. Y. Hein, T. Geiger, M. Mann, J. Cox, The Perseus computational platform for comprehensive analysis of (prote)omics data. *Nat. Methods* **13**, 731–740 (2016).

80. M. Sakai, T. D. Troutman, J. S. Seidman, Z. Ouyang, N. J. Spann, Y. Abe, K. M. Ego, C. M. Bruni, Z. Deng, J. C. M. Schlachetzki, A. Nott, H. Bennett, J. Chang, B. T. Vu, M. P. Pasillas, V. M. Link, L. Texari, S. Heinz, B. M. Thompson, J. G. McDonald, F. Geissmann, C. K. Glass, Liver-derived signals sequentially reprogram myeloid enhancers to initiate and maintain Kupffer cell identity. *Immunity* **51**, 655–670.e8 (2019).

81. S. Heinz, C. Benner, N. Spann, E. Bertolino, Y. C. Lin, P. Laslo, J. X. Cheng, C. Murre, H. Singh, C. K. Glass, Simple combinations of lineage-determining transcription factors prime cis-regulatory elements required for macrophage and B cell identities. *Mol. Cell* **38**, 576–589 (2010).

82. A. Dobin, C. A. Davis, F. Schlesinger, J. Drenkow, C. Zaleski, S. Jha, P. Batut, M. Chaisson, T. R. Gingeras, STAR: Ultrafast universal RNA-seq aligner. *Bioinformatics* **29**, 15–21 (2013).

83. M. I. Love, W. Huber, S. Anders, Moderated estimation of fold change and dispersion for RNA-seq data with DESeq2. *Genome Biol.* **15**, 550 (2014).

84. Y. Zhou, B. Zhou, L. Pache, M. Chang, A. H. Khodabakhshi, O. Tanaseichuk, C. Benner, S. K. Chanda, Metascape provides a biologist-oriented resource for the analysis of systems-level datasets. *Nat. Commun.* **10**, 1523 (2019).

85. H. Pagès, M. Carlson, S. Falcon, N. Li, AnnotationDbi: Manipulation of SQLite-based annotations in Bioconductor. R package version 1.66.0. (2024); https://bioconductor.org/packages/AnnotationDbi

86. H. Wickham, M. Averick, J. Bryan, W. Chang, L. M. Gowan, R. François, G. Grolemund, A. Hayes, L. Henry, J. Hester, M. Kuhn, T. Pedersen, E. Miller, S. Bache, K. Müller, J. Ooms, D. G. Robinson, D. Seidel, V. Spinu, K. Takahashi, D. Vaughan, C. Wilke, K. H. Woo, H. Yutani, Welcome to the tidyverse *J. Open Source Softw.* **4**, 1686 (2019).

87. T. Wu, E. Hu, S. Xu, M. Chen, P. Guo, Z. Dai, T. Feng, L. Zhou, W. Tang, L. Zhan, X. Fu, S. Liu, X. Bo, G. Yu, Yu, clusterProfiler 4.0: A universal enrichment tool for interpreting omics data. *Innovation (Camb)* **2**, 100141 (2021).

88. G. Yu, Q. Y. He, ReactomePA: An R/Bioconductor package for reactome pathway analysis and visualization. *Mol. Biosyst.* **12**, 477–479 (2016).

89. H. Wickham, *ggplot2: Elegant Graphics for Data Analysis*, Use R! (Springer, 2nd Ed., 2016).

90. C. Arshadi, U. Gunther, M. Eddison, K. I. S. Harrington, T. A. Ferreira, SNT: A unifying toolbox for quantification of neuronal anatomy. *Nat. Methods* **18**, 374–377 (2021).

91. T. A. Ferreira, A. V. Blackman, J. Oyrer, S. Jayabal, A. J. Chung, A. J. Watt, P. J. Sjostrom, D. J. van Meyel, Neuronal morphometry directly from bitmap images. *Nat. Methods* **11**, 982–984 (2014).

92. J. Cox, M. Y. Hein, C. A. Luber, I. Paron, N. Nagaraj, M. Mann, Accurate proteome-wide label-free quantification by delayed normalization and maximal peptide ratio extraction, termed MaxLFQ. *Mol. Cell. Proteomics* **13**, 2513–2526 (2014).
